# Development and preliminary evaluation of a 90 K Axiom® SNP array for the allo-octoploid cultivated strawberry *Fragaria**** × ****ananassa*

**DOI:** 10.1186/s12864-015-1310-1

**Published:** 2015-03-07

**Authors:** Nahla V Bassil, Thomas M Davis, Hailong Zhang, Stephen Ficklin, Mike Mittmann, Teresa Webster, Lise Mahoney, David Wood, Elisabeth S Alperin, Umesh R Rosyara, Herma Koehorst-vanc Putten, Amparo Monfort, Daniel J Sargent, Iraida Amaya, Beatrice Denoyes, Luca Bianco, Thijs van Dijk, Ali Pirani, Amy Iezzoni, Dorrie Main, Cameron Peace, Yilong Yang, Vance Whitaker, Sujeet Verma, Laurent Bellon, Fiona Brew, Raul Herrera, Eric van de Weg

**Affiliations:** USDA-ARS, NCGR, Corvallis, OR USA; University of New Hampshire, Durham, NH USA; Washington State University, Pullman, WA USA; Affymetrix, Santa Clara, CA USA; Michigan State University, East Lansing, MI USA; Wageningen-UR Plant Breeding, Wageningen, The Netherlands; IRTA-Center for Research in Agricultural Genomics CSIC-IRTA-UAB-UB, Barcelona, Spain; Fondazione Edmund Mach, Research and Innovation Centre, San Michele all’Adige, 38010 TN Italy; IFAPA-Centro de Churriana, Málaga, Spain; INRA, UMR BFP, Bordeaux, France; University of Florida, GCREC, Wimauma, FL USA; Affymetrix UK Ltd, Wooburn Green, High Wycombe, UK; Instituto Ciencias Biologicas, Universidad de Talca, Talca, Chile

**Keywords:** *Fragaria*, Genotyping array, Plant breeding, Polyploidy, Strawberry, Single nucleotide polymorphism, Reduced ploidy

## Abstract

**Background:**

A high-throughput genotyping platform is needed to enable marker-assisted breeding in the allo-octoploid cultivated strawberry *Fragaria* × *ananassa*. Short-read sequences from one diploid and 19 octoploid accessions were aligned to the diploid *Fragaria vesca* ‘Hawaii 4’ reference genome to identify single nucleotide polymorphisms (SNPs) and indels for incorporation into a 90 K Affymetrix® Axiom® array. We report the development and preliminary evaluation of this array.

**Results:**

About 36 million sequence variants were identified in a 19 member, octoploid germplasm panel. Strategies and filtering pipelines were developed to identify and incorporate markers of several types: di-allelic SNPs (66.6%), multi-allelic SNPs (1.8%), indels (10.1%), and ploidy-reducing “haploSNPs” (11.7%). The remaining SNPs included those discovered in the diploid progenitor *F. iinumae* (3.9%), and speculative “codon-based” SNPs (5.9%). In genotyping 306 octoploid accessions, SNPs were assigned to six classes with Affymetrix’s “SNPolisher” R package. The highest quality classes, *PolyHigh Resolution* (*PHR*), *No Minor Homozygote* (*NMH*), and *Off-Target Variant* (*OTV*) comprised 25%, 38%, and 1% of array markers, respectively. These markers were suitable for genetic studies as demonstrated in the full-sib family ‘Holiday’ × ‘Korona’ with the generation of a genetic linkage map consisting of 6,594 *PHR* SNPs evenly distributed across 28 chromosomes with an average density of approximately one marker per 0.5 cM, thus exceeding our goal of one marker per cM.

**Conclusions:**

The Affymetrix IStraw90 Axiom array is the first high-throughput genotyping platform for cultivated strawberry and is commercially available to the worldwide scientific community. The array’s high success rate is likely driven by the presence of naturally occurring variation in ploidy level within the nominally octoploid genome, and by effectiveness of the employed array design and ploidy-reducing strategies. This array enables genetic analyses including generation of high-density linkage maps, identification of quantitative trait loci for economically important traits, and genome-wide association studies, thus providing a basis for marker-assisted breeding in this high value crop.

**Electronic supplementary material:**

The online version of this article (doi:10.1186/s12864-015-1310-1) contains supplementary material, which is available to authorized users.

## Background

A central goal of several international consortia led by the RosBREED project [1] has been to establish high-throughput genotyping platforms for five rosaceous crops: peach, apple, sweet and sour cherry, and strawberry, to facilitate marker-assisted breeding in these economically and nutritionally important crops. This goal has been realized in part through the development of three SNP (single nucleotide polymorphism) arrays: a 9 K whole genome scanning array for peach [2], an 8 K apple and 1 K pear array [3,4], and a 6 K array for cherry [5]. These three projects utilized the Illumina® Infinium® genotyping platform. To date, these arrays have been used for the generation of linkage maps [4,6-11], evaluation of the quality of physical maps [12], fine mapping and validation of quantitative trait loci (QTL) [9,13], elucidation of marker-trait associations [10,14], genome-wide association studies [15], genomic selection studies [16], validation of pedigrees and verification of trueness to type of breeding lines and accessions [17], and for design of the future generation of arrays [18]. Thus, these three arrays have had broad utility for the Rosaceae genomics and genetics research and breeding communities.

### Polyploidy is challenging for SNP discovery and genotyping

Polyploidy and whole genome duplication have long been recognized as major components of both genome and species evolution [19], and are widely evident in Rosaceae genera including *Fragaria*, *Malus*, *Prunus*, and *Rubus*. Polyploidy is prevalent in many plant families, and it is estimated that 50 to 70% of flowering plants are polyploids [20]. In addition to the plethora of examples represented by polyploid complexes, all the sequenced plant genomes previously considered as “diploids” (e.g., apple [21], rice [22], poplar [23] or grape [24]) have revealed superimposed traces of past genome duplication events [25]. Polyploidization is usually followed by processes of genomic and/or chromosomal diploidization, such as homoeolog loss [26], divergence of homoeolog expression leading to bias that may favor one of the subgenomes [27], and establishment of preferential, bivalent pairing [28].

Marker SNPs are DNA sequence variants at orthologous sites within or between individuals. In array development projects, SNPs are discovered by alignment of sequences from a detection panel to a reference genome. In diploid species projects, such as for sweet cherry and peach, marker SNPs need only be distinguished from variants at paralogous sites within the diploid genome. However, as reviewed by Kaur *et al*. [29], in allopolyploid plants, paralogy is possible both within and between homoeologous subgenomes, thus complicating the differentiation of marker SNPs from nuisance paralogous variants. Of particular concern and interest in allopolyploids are homoeologous sequence variants (HSVs), which are variants occurring at corresponding reference coordinates but between, rather than within, subgenomes [30]. Following Kaur *et al.* [29], HSVs are distinct from type 1 and type 2 paralogous sequence variants (PSVs), which occur, respectively, at non-identical reference coordinates within and between subgenomes. To date, high-density marker platforms have been developed for very few polyploid crops: auto-tetraploid potato [31], allo-tetraploid sour cherry [5], rose [32], and oilseed rape [33,34], as well as the more complex allo-hexaploid wheat [35]. To address the challenges of SNP detection in polyploids, the scientific communities in some of these important crops have formed international research and development consortia, thereby facilitating the development of a 9 K Infinium array in wheat [35] and the 7 K [33] and 60 K Infinium arrays [34] in allo-tetraploid oilseed rape.

### Polyploidy in the cultivated strawberry

The cultivated strawberry, *Fragaria* × *ananassa* (Duch.), is an allo-octoploid (2*n* = 8*x* = 56) species that arose from a chance hybridization in a European botanical garden in the mid-1700s between representatives of the octoploid species *F. chiloensis* (Mill.) and *F. virginiana* (Mill.) [36]. An allo-polyploid AAA'A'BBB'B' model comprising four differentiated subgenomes was proposed for the *Fragaria* octoploids by Bringhurst [37], and is consistent with reports of full disomic inheritance in marker-based linkage maps [38,39]. However, a definitive model of subgenome composition for the cultivated strawberry and its progenitor species has not yet been established, nor has it been confirmed that a common model would be applicable to all octoploid germplasm. In this study, we adopted the simplifying assumption that the cultivated strawberry genome composition conforms to an allo-polyploid model of four distinct subgenomes: AABBCCDD.

Early cytogenetic and cross-ability studies and subsequent molecular analyses implicated diploid *F. vesca* as a likely progenitor and A-subgenome donor to the cultivated strawberry and its octoploid species ancestors, and phylogenetic analyses based on almost complete chloroplast sequences of 21 *Fragaria* species and subspecies identified the western North American *F. vesca* subsp. *bracteata* as the likely chloroplast genome donor [40]. A reference genome sequence of the A-related *F. vesca* subsp. *vesca* accession ‘Hawaii 4’ (PI551572, National Clonal Germplasm Repository, Corvallis, OR) has been published [41]. Diploid *Fragaria iinumae* has been suggested as a second genome donor to the octoploids, based on phylogenetic analyses of low-copy nuclear loci [42,43], and has been shown to uniquely share a robust mitochondrial marker with the octoploids [44].

### SNP detection in strawberry

A preliminary attempt to identify SNPs in strawberry from transcriptome sequences resulted in a low validation rate of 9% in a Mendelian transmission test, likely caused by misidentification and coincidence with HSVs [29]. To address the predicted challenges of a low rate of validation and difficulties in accurate automated genotyping by existing software programs for analysing array data (described for hexaploid wheat by Akhunov *et al*. [45]), the International Strawberry Consortium developed multiple approaches for SNP discovery and array design in strawberry, which are described herein. To simplify genotype scoring and enhance the accuracy of automated genotyping, we have developed standard di-allelic SNP and indel-based markers and have assessed the potential of using multi-allelic SNPs. In addition, we devised an innovative new class of markers called “haploSNPs” as the basis for achieving a technical reduction in ploidy and thereby diminishing the problem of cluster compression associated with SNP array genotype calling in polyploids. A novel, non-discovery-based SNP marker development strategy was also explored. Finally, a set of array SNPs was developed specifically for mapping purposes in the ancestral diploid, *F. iinumae*. Herein is reported the development and preliminary evaluation of the first high-throughput SNP genotyping platform for strawberry: a 90 K SNP array named IStraw90 (for International Strawberry 90 K) based on the Affymetrix Axiom platform.

## Methods

### Sequence resources

The genomes of 19 octoploid and six diploid strawberry accessions were sequenced to serve as resources for SNP discovery and interpretation (Table 1). The octoploid germplasm Global Discovery Panel (GDP) included: 1) a diverse sampling of 15 *F.* ×*ananassa* accessions that comprised six cultivars (Holiday, Korona, Emily, Fenella, Sweet Charlie, and Winter Dawn), two breeding selections (CA65.65.601 and NH-SB480), six F_1_ progeny (the “HolKor” seedlings) from ‘Holiday’ × ‘Korona’, and one F_2_ generation progeny plant from ‘Dover’ × ‘Camarosa’; and 2) one and three accessions respectively of the ancestral octoploids *F. virginiana* and *F. chiloensis* (Table 1). The diploids included three representatives of *F. vesca*, one of *F. mandshurica*, and two of *F. iinumae* (Table 1). Of the latter, accession F1D is an intraspecific hybrid that is being used as a parent in an *F. iinumae* linkage mapping project (Mahoney *et al*., manuscript in preparation).

**Table 1:** **Strawberry accessions and sequence data used for variant discovery in the respective filtration panels**

**Name**	**Taxon**	**NCBI SRX numbers**	**Total trimmed reads (Million)**	**Mean coverage depth (** ***x*** **)**	**GDP**	**HD-16**	**HD-20**
**‘Winter Dawn’**	*F.* ×*ananassa*	SRX651592	394.5	48.7	√	√	√
**‘Sweet Charlie’**	*F.* ×*ananassa*	SRX651582	407.8	37.0	√	√	√
**‘Fenella’**	*F.* ×*ananassa*	SRX651547	397.7	34.9	√	√	√
**HolKor 2321**	*F.* ×*ananassa*	SRX651548	221.6	32.3	√	√	√
**‘Emily’**	*F.* ×*ananassa*	SRX651546	400.4	31.2	√	√	√
**HolKor 2637** ^**1**^	*F.* ×*ananassa*	SRX651574	220.1	30.0	√	√	√
**HolKor 2557**	*F.* ×*ananassa*	SRX651553	208.6	26.0	√	√	√
**HolKor 2549**	*F.* ×*ananassa*	SRX651551	204.7	24.9	√	√	√
**Dover × Camarosa F**_**2**_ **_34** ^**2**^	*F.* ×*ananassa*	SRX651599	76.9	19.7	√	√	-
**HolKor 2637** ^**3**^	*F.* ×*ananassa*	SRX651567	113.4	16.7	√	√	-
**HolKor 2580**	*F.* ×*ananassa*	SRX651558	192.2	15.1	√	-	-
**‘Korona’**	*F.* ×*ananassa*	SRX651580	194.2	14.2	√	-	-
**HolKor 2529**	*F.* ×*ananassa*	SRX651549	125.1	5.7	√	-	-
**‘Holiday’**	*F.* ×*ananassa*	SRX651579	123.1	4.6	√	-	-
**CA65.65-601**	*F.* ×*ananassa*	SRX651545	126.1	4.3	√	-	-
**NH SB480** ^**4**^	*F.* ×*ananassa*	-	107.9	2.7	√	-	-
**CFRA 1992 (BC6)**	*F. virginiana*	SRX651527	101.8	2.0	√	-	-
**CFRA 1691**	*F. chiloensis*	SRX651521	142.1	5.6	√	-	-
**CFRA 743**	*F. chiloensis*	SRX651520	397.5	4.1	√	-	-
**Fc** ^**4**^	*F. chiloensis*	-	359.7	4.0	√	-	-
**CFRA 480 (‘Yellow Wonder’)** ^**5**^	*F. vesca*	SRX651526	38.3	9.5	-	-	-
**CFRA 1984 (‘Pawtuckaway’)** ^**5**^	*F. vesca*	SRX651525	39.3	9.1	-	-	-
**CFRA 985 (‘Baron Solemacher’)** ^**5**^	*F. vesca*	SRX651524	30.9	6.6	-	-	-
**CFRA 1947** ^**6**^	*F. mandshurica*	SRX651523	31.8	7.1	-	-	-
**CFRA 1849** ^**6**^	*F. iinumae*	SRX651522	35.5	4.6	-	-	-
**F1D** ^**4**^	*F. iinumae*	-	171.0	36.0	-	-	-

^1^Sequenced at the Center for Genome Research and Biocomputing at Oregon State University.

^2^150 bp paired-end sequencing and use of GAIIx for sequencing.

^3^Sequenced by Illumina Inc.

^4^Sequences provided upon request from Thomas Davis.

^5^Illumina index adaptors were added and samples were pooled in equimolar amounts for sequencing in one lane.

^6^80 bp paired-end sequencing with GAIIx.

The octoploid Global Discovery Panel (GDP) and *F. iinumae* F1D sample were used for variant discovery. Filtration subpanels consisted of octoploid HD-16 and HD-20 subpanels described in the Methods. Total trimmed reads, mean coverage depth and NCBI SRX numbers are listed. Note that the HolKor 2637 seedling was sequenced twice, and so is listed twice in this table.

### Validation set

The strawberry accessions chosen to validate usefulness of the array consisted of the following: 306 octoploid *F.* ×*ananassa* breeding accessions and cultivars (Table 2, Additional file 1); 51 “non-ananassa” octoploid accessions; three widely studied accessions of diploid *F. vesca*; and a pedigree-connected population of diploid *F. iinumae* that included crossing parents J17 and J4, their first generation hybrid F1D, and 21 second generation ‘F2D’ progeny. The 306 octoploid *F.* ×*ananassa* samples (Table 2, Additional file 1) encompassed: all members of the GDP, including four replicate samples of ‘Korona’ and two of ‘Holiday’; one large mapping population of 80 offspring (‘Holiday’ × ‘Korona’); three small mapping populations (20–40 offspring); and founding parents and progeny of public breeding programs in the U.S. including the University of Florida, Michigan State University, and the USDA-ARS Corvallis programs. The 51 “non-ananassa” octoploid accessions included 10 parents and progeny from a *F.* ×*ananassa* reconstruction population [46] named FVC, and 41 individuals of multiple pedigree-connected families from the New Hampshire breeding program (UNH_1 through UNH_41).

**Table 2:** **Summary of strawberry samples (SNP Validation Set) evaluated with the array**

**Validation set/Category**	**Number of individuals**
***F. ×ananassa*** **GDP members**	**13**
**University of Florida selections**	**25**
**Cultivars, selections and parents of breeding populations**	**43**
**‘Holiday’ replicates**	**1**
**‘Korona’ replicates**	**3**
**Mapping populations (4)**	
‘Holiday’ × ‘Korona’	**74**
‘Tribute’ × ‘Honeoye’	**26**
‘Capitola’ × CF1116	**20**
‘Redgauntlet’ × ‘Hapil’	**40**
***F.*** **×** ***ananassa*** **octoploid breeding populations (7)**	
USDA-ARS Corvallis ORUS_3278	**10**
USDA-ARS Corvallis ORUS_3315	**10**
USDA-ARS Corvallis ORUS_3316	**10**
USDA-ARS Corvallis ORUS_3323	**6**
USDA-ARS Corvallis ORUS_3326	**5**
Michigan State University MSU_9-18	**10**
Michigan State University MSU_9-9	**10**
***“*** **Non-ananassa** ***”*** **octoploid breeding populations (2)**	**10**
ORUS/MSU FVC	
University of New Hampshire UNH_1-41	**41**
***F. vesca*** **cultivars**	**3**
***F. iinumae*** **diploid pedigreed population (F2)**	**24**
**Total**	**384**

### Library preparation and sequencing

With the exception of the *F. iinumae* F1D and HolKor 2637 samples, DNA for Illumina library preparation was extracted from either fresh or freeze-dried unfolded leaves with the E-Z 96® Plant DNA Kit (Omega Bio-Tek) [47] and quantified with the Quant-iT™ PicoGreen® Assay (Life Technologies) using a Victor multiplate reader (Perkin Elmer Inc.). The F1D DNA was isolated using a CTAB miniprep method [48]. The HolKor DNA was isolated as previously described [38]. With the exception of HolKor 2637, library preparations were performed with either the Illumina’s TruSeq DNA v2 kit (Illumina Inc.) or using a modified version of Illumina’s Paired-End protocol and non-Illumina enzymes, primers and adaptors (Table 1). HolKor 2637 library preparation and sequencing were performed by Illumina, Inc. Library preparation for each strawberry sample is described in Additional file 2. The sequence data are deposited in the NCBI Sequence Read Archive (SRA) as BioProject PRJNA254712 with SRA Experiment accession (SRX) numbers as listed in Table 1.

### Sequence alignment

A Variant Call Format (VCF) file was generated from each of the Illumina short read data sets listed in Table 1. The fastx_barcode_splitter.pl script from the fastx_toolkit (http://hannonlab.cshl.edu/fastx_toolkit/) was used to separate reads in multiplexed *F. vesca* accessions (‘Pawtuckaway’, ‘Yellow Wonder’ and ‘Baron Solemacher’). Adaptor sequences and low-quality ends were removed from the raw reads using *cutadapt* with filtering set at a phred compatible score of 20 and a minimum length of 26 to keep the read [49]. The Burrows-Wheeler aligner (BWA), with default arguments [50], was used to align the resulting reads to the *F. vesca* ‘Hawaii 4’ v1.1 reference genome [41,51,52]. The BWA *aln* command was used with both forward and reverse reads from each panel member to produce separate forward and reverse sequence alignment index (.sai) files. The BWA *sampe* command was used to incorporate the .sai files into sequence alignment maps (.sam) for paired-end reads. The *samse* command was used for incorporating reads with a missing mate-pair. The sequence alignment map (SAM) files were then converted into binary alignment map (BAM) files using the SAMTools *view* command. The BAM files were sorted using the SAMTools *sort* command, then indexed with the SAMTools *index* command. Duplicate reads that had been generated during library preparation as an artifact of the PCR enrichment process were removed using the SAMTools *rmdups* command [53]. The BAM files were reformatted using the Picard-tools *reheader* command, then subjected to local realignment around indel sites using the genome analysis toolkit GATK [54] to remedy alignment issues associated with small indel polymorphisms occurring near the beginnings and/or ends of the short reads. From the resulting “improved” BAM files, read depth per coordinate was calculated for each accession in the discovery panel using the SAMTools *depth* command, and mean coverage depth per accession was calculated from the resulting coverage files (Table 1). Variant calling was achieved using FreeBayes (http://arxiv.org/abs/1207.3907) to produce VCF files, one for each of the sequenced accessions. The original Illumina sequences and resulting alignment files in BAM format are available (ftp://ftp.bioinfo.wsu.edu/projects/RosBREED/strawberry/).

### Variant discovery and filtration panels

Variant discovery was conducted separately in the octoploid germplasm panel versus in diploid *F. iinumae* hybrid F1D. For variant discovery at the octoploid level, the VCF files from all members of the GDP were employed. As noted in Additional file 2, HolKor 2637 was sequenced twice and was therefore represented by two VCF files, so the GDP of 19 octoploid accessions was represented by 20 VCF files. For subsequent filtration procedures, two GDP subpanels were defined. The HD-16 subpanel consisted of the ten data sets with a minimum of 16× genome coverage (Table 1), and as such included both of the HolKor 2637 samples. The HD-20 subpanel consisted of the eight data sets with a minimum of 20× coverage (Table 1), and therefore excluded the lower-coverage HolKor 2637 sample.

### Variant filtering in octoploids

**Figure 1:**
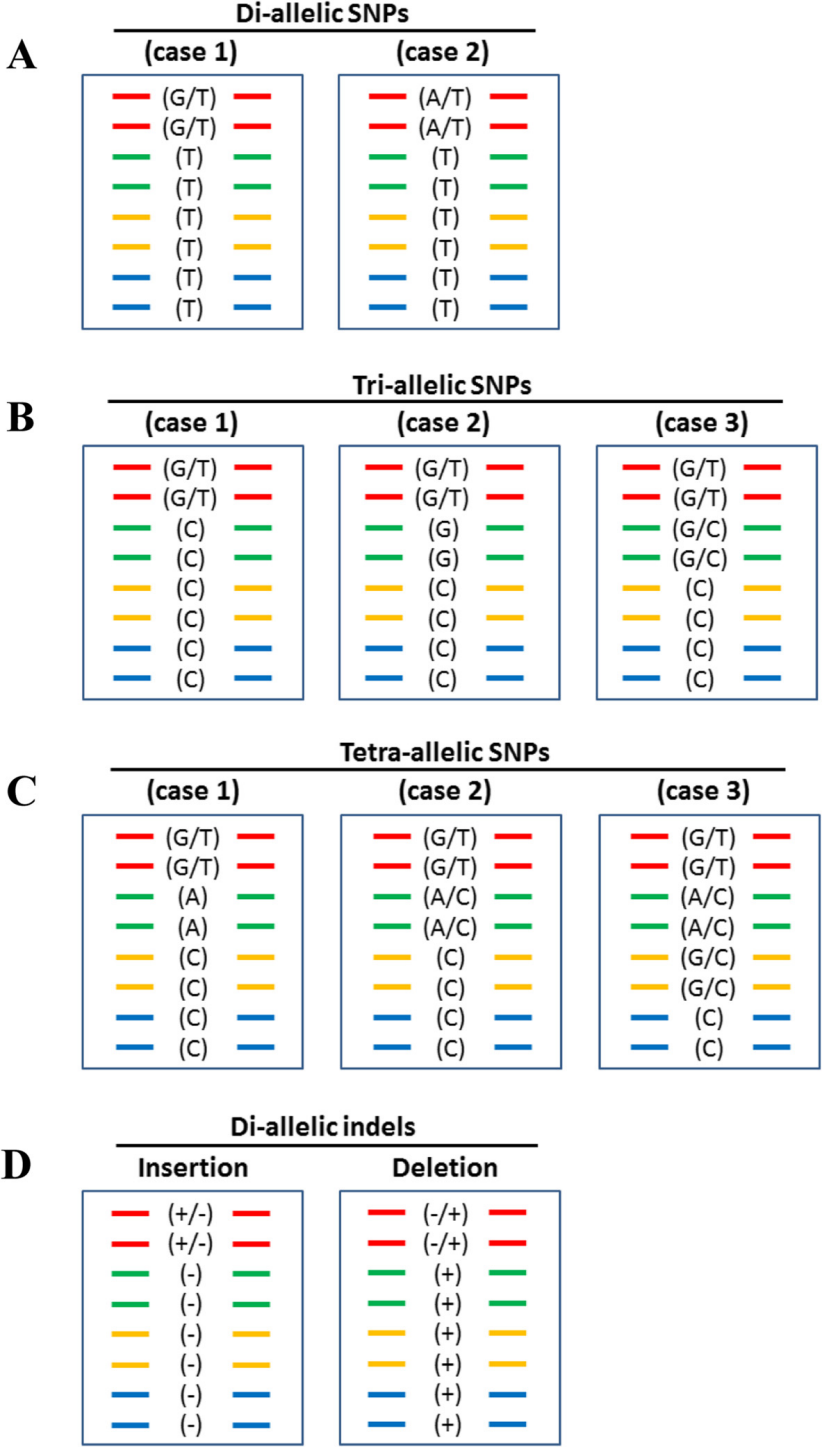
**Allelic configurations of SNP (di-allelic and multi-allelic) and indel markers in an octoploid.** Panel **A)** Di-allelic SNPs: To qualify as di-allelic, only two alleles can be detected at the site. The “marker allele” is present only in one subgenome (the marker subgenome), within which it can be homozygous present, heterozygous, or homozygous absent. In case 1 a single probe can be used to interrogate the marker because the indicated polymorphism is neither A/T nor G/C. In case 2 two probes must be used because the indicated polymorphism is an A/T (also true for a G/C polymorphism). Panels** B** and **C)** Multi-allelic SNPs: More than two alleles are represented at the site. Three distinctive cases are shown for tri-allelic (Panel **B)** and for tetra-allelic sites (Panel **C)**. In tri-allelic case 1 the marker polymorphism is G/T, while there is a C at the same site in the background subgenomes. Genotyping of this marker would require two probes. In case 2 the marker polymorphism is G/T, with a background G in one subgenome and a background C in the others. Genotyping of this marker would require two probes. In case 3 there are two marker polymorphisms, a G/T in one subgenome and a G/C in another, while there is a C at the site in the background subgenomes. Three probes and a non-standard analysis algorithm are needed for this polymorphism. Genotyping of case 3 tri-allelic markers, and of tetra-allelic markers (Panel **C)** is currently not possible. Panel** D)** Di-allelic indels: Only two alleles are represented at the site. Although they are genomic insertions and deletions, the indel polymorphisms are genotyped as SNPs, and various probing strategies may be employed depending upon the sequence characteristics within and immediately adjacent to the indel.

**Figure 2:**
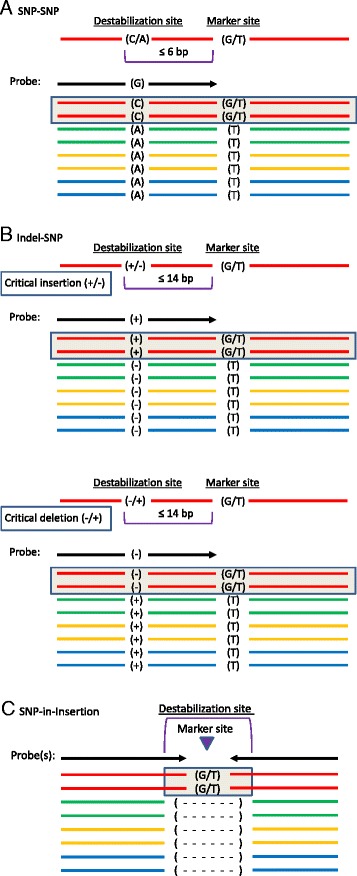
**Representation of the three haploSNP categories consisting of SNP-SNP (A), Indel-SNP (B), and SNP-in-Insertion (C).** A) In the SNP-SNP **(A)** and indel-SNP **(B)** strategies the “critical form” of the destabilizing site, to which the probe is targeted, must be coupled to the SNP marker allele. Due to its asymmetric nature, a SNP-SNP or indel-SNP site can be probed only on one strand. A single probe is employed if the marker polymorphism is not A/T or G/C, while two probes are required if it is A/T or G/C. In the SNP-SNP strategy, the destabilizing SNP site must be present within 6 bp of the marker SNP site, while in the indel-SNP strategy, the destabilizing indel site must be present within 14 bp of the marker SNP site. In relation to the background alleles, the critical form of an indel destabilization site **(B)** can be either an insertion or a deletion. In the SNP-in-Insertion **(C)** strategy, the probe is expected to anneal only to the insertional form of an indel, and to interrogate a SNP polymorphism that resides within the insertion in one subgenome. A SNP-in-Insertion site can be probed on either or both strands.

The GDP VCF files were entered into various filtering pipelines (Additional files 3, 4, 5, 6, 7 and 8) aimed at discovering marker candidates of several types: di-allelic SNPs (Figure 1A; Additional file 3), multi-allelic SNPs (mSNPs) (Figure 1B-C; Additional file 4), di-allelic indels (Figure 1D, Additional file 5), and three categories of haploSNPs (Figure 2A-C; Additional files 6, 7 and 8). The term “haploSNP” denotes the coupling of two variants: (1) a marker SNP and (2) a closely adjacent HSV SNP or indel that provides a critical “destabilization site”, which is intended to confer subgenomic exclusivity and thereby achieve technical ploidy reduction at the respective site of probe hybridization.

Key filters or filtration steps that were included in more than one pipeline are described as follows and exemplified in Additional files 3, 4, 5, 6, 7, 8 and 9. In-house Python scripts were written to implement the various filtration pipelines. The Minimum Variant Read Count filter excluded variants that occurred in less than three (for SNPs) or two (for indels) reads when summed across all GDP VCF files. Its purpose was to remove variants likely due to sequencing errors. The Minimum Presence filter excluded SNP variants not represented in at least two members of the indicated germplasm panel (GDP) or subpanel (HD-16, or HD-20), while the Minimum Absence filter excluded SNP or indel variants not absent in at least two members of the indicated germplasm panel (GDP) or subpanel (HD-16, or HD-20). In combination, the Minimum Presence and Absence filters were intended to ensure that identified variants were polymorphic among germplasm panel members and therefore would constitute markers rather than HSVs.

The A/T-G/C filter was used to exclude A/T and G/C variants in the di-allelic SNP and codon-based pipelines, on the basis that such variants require the use of multiple probes in the Axiom platform. The CDS or Genic filters were employed to exclude variants that were not located in coding sequence or in coding plus intron sequence, respectively, as determined by gene models for the *F. vesca* v. 1.0 reference genome [41]. The marker candidates identified as residing on ‘Hawaii 4’ linkage group 0 (which consisted of contigs not assigned to linkage groups 1 through 7), were ultimately excluded from consideration because they could not be subjected to CDS confirmation.

The UpSafe-DownSafe filters were used to assure that the regions immediately upstream (UpSafe) or downstream (DownSafe), or both upstream and downstream (BothSafe), of a marker variant were free of any other variants across the GDP. This filtration step was intended to maximize the likelihood that probe sites were conserved across diverse germplasm.

Di-allelic and multi-allelic SNP (mSNP) candidates and di-allelic indel candidates were identified as described in Additional files 3, 4, and 5, respectively. The di-allelic SNP and mSNP pipelines identified candidate sites lacking polymorphisms within 24 bp upstream and downstream of the variant site, while in the di-allelic indel pipeline the upstream and downstream exclusion intervals were 24 bp and 30 bp, respectively; the extra 6 bp being added downstream because the 3–6 bp size of the candidate indels contributed to the nominal downstream distance from the candidate coordinate site.

### HaploSNP strategies and filters

As defined above and depicted in Figure 2, ploidy-reducing haploSNPs consist of two closely coupled or overlapping variant sites: a marker site and a “destabilization site”. The marker site is the site of the SNP variant that is to be genotyped and is intended to be polymorphic within a single subgenome. The “destabilization site” is defined as the site of a SNP (Figure 2A) or indel (Figure 2B, C) identified as polymorphic among subgenomes (i.e., an HSV as defined by Kaur *et al. *[29]), and non-polymorphic within the subgenome containing the marker variant. The marker SNP site is intended to be interrogated by a probe that targets the “critical” form of the HSV: i.e., the form that is coupled with the marker variant. Thus, the probe is expected to hybridize to the subgenome that contains the marker variant (Figure 2A, B & C), but fail to hybridize to one or more of the subgenomes that lack the marker variant (thereby achieving ploidy reduction). For each of the three haploSNP categories, the degree of achieved ploidy reduction depends on whether the critical form of the target site occurs in only one (reduction to diploid), two (reduction to tetraploid), or three (reduction to hexaploid) subgenomes.

Within the haploSNP category, three strategies were explored, each requiring a distinct filtration pipeline. In the SNP-SNP strategy (Additional file 6), the destabilizing HSV was a SNP residing within 6 bp of the marker variant site (Figure 2A). In the Indel-SNP strategy (Additional file 7), the destabilizing HSV was a 4-to-6 bp indel residing within 14 bp of the marker variant site (Figure 2B). In the SNP-in-Insertion strategy (Additional file 8), the HSV was an insertion (relative to other subgenomes) of 3–6 bp which contained within it the marker SNP site (Figure 2C). In each of the three haploSNP strategies, no variant other than the destabilizing HSV was permitted within the 24 bp region delimited by the marker SNP site and containing the destabilization site.

In each of the three haploSNP pipelines, the set of ~36 million variants was initially mined for sites that could serve as critical destabilization sites, after which the pipeline proceeded to the identification of neighbouring marker SNP candidates. The remaining pipeline steps were aimed at identifying suitable pairings of marker SNP and destabilization site candidates, and confirming that all instances of the marker SNP were coupled to the targeted form of the destabilization site.

### Variant discovery in diploid *F. iinumae*

For diploid *F. iinumae*, variants were initially discovered by specifically mining the *F. iinumae* F1D VCF file (Additional file 9). SNPs were sought that were heterozygous in F1D and would therefore be expected to segregate in the derived F2D mapping population. Many of the filtration procedures used in di-allelic SNP discovery in the octoploid GDP were also employed for SNP discovery in F1D as described in Additional file 9. Importantly, in the discovery of F1D SNPs, the Upsafe-DownSafe filter employed all of the octoploid GDP VCFs as well as the F1D VCF to maximize the likelihood that F1D-based probe sites would also be conserved in octoploid germplasm.

### Non-discovery-based approach

Finally, a novel, non-discovery-based approach was explored, and is referred to as the “codon-based” approach. Here, coding sequences (CDS) that were widely distributed across the seven ‘Hawaii 4’ linkage groups, as determined from gene models for the *F. vesca* v1.0 reference genome [41], were mined for proline codons. Probes were then designed to interrogate the third positions of these 4-fold degenerate codons without regard to or reliance on prior variant discovery. These codon-based SNP candidates were purely speculative, and were included in our study to test the hypothesis that SNPs could be discovered in useful numbers through the array genotyping process itself without relying on extensive, prior sequencing of germplasm panels.

### Affymetrix filtering and axiom array design and construction

For each marker type, a list of candidate sites was submitted to Affymetrix for further distillation into a final set of sites that met quality criteria for representation on the array. Then, depending upon the marker category, the nature of the polymorphism and other criteria as detailed below, an appropriate probing strategy was defined for each site, utilizing from one to eight distinct probe sequences on the array.

Candidate markers were chosen for array inclusion in the following order of priority: (1) markers expected to provide ploidy reduction, including mSNPs and haploSNPs, (2) markers from the remaining categories identified from the octoploid VCFs (di-allelic SNPs and di-allelic indels), (3) F1D SNPs, and (4) codon-based SNPs that met the threshold criteria for predicting reproducibility of markers based on Affymetrix’ in silico design scores.

To prioritize probe sets for polymorphisms, the flanking sequences of each candidate marker site were analyzed by the Affymetrix Bioinformatics Team for the following characteristics:

1. Existence of other polymorphisms within 24 bases that align with sequence adjacent to the targeted polymorphism site. Probe design was classified as: “not recommended” when one or more polymorphisms were found within 20 bases, or more than 2 polymorphisms within 24 bases; “neutral” when one or two polymorphisms were found between 20 and 24 bases, and; “recommended” if no polymorphisms were found. In contrast to the previously applied UpSafe/DownSafe filters, which utilized comparisons of Illumina reads aligned to the reference genome, the Affymetrix filter relied on comparisons among the flanking sequences of the submitted marker candidates and was intended to preclude interrogation of paralogous marker sites.
2. High sequence similarity to the rest of the genome as measured by a count of all of the 16-mer substrings of the probe found in the genome. Probe design was classified as: “not recommended” when more than 300 matches of 16-mers were found in the genome.
3. Predicted probe quality based on a random forest model trained on the performance of 700,000 human SNPs. Probe design was classified as: “not recommended” for a score below 0.4; “neutral” if the score was between 0.4 and 0.7, and “recommended” if the score exceeded 0.7.

For the standard (di-allelic) SNPs and indels, including F1D and codon-based SNPs, only recommended probes were used. For the remaining SNP categories (mSNPs and haploSNPs), recommended and neutral probes were included in order to fully populate the array. Multi-allelic single-site polymorphisms were interrogated by designing all four bases at that position.

The array was built using the Axiom myDesign™ custom genotyping platform. Markers were designed with two replicates on either the forward or reverse strand. In a subset of the polymorphisms from most SNP categories except for SNP-SNPs and indel-SNPs, both strands were tiled, given they passed the reproducibility threshold. The resulting design included 85,663 polymorphisms from the octoploid genome, along with 3,751 target sites from the diploid *F. iinumae* genome, and 5,648 speculative (codon-based) sites (Table 3). The A/C, A/G, C/T and G/T polymorphisms were interrogated with two replicates with a single probe while A/T and C/G SNPs were interrogated with two replicates each of two different probes. The mSNPs were interrogated with two replicates of four distinct probes. The total number of target sites of 95,062 was interrogated with 138,099 probesets (Table 3, Additional file 10). After accounting for all of the replicated probes the total number of 6-micron square features on the chip dedicated to strawberry polymorphisms was 275,636.

**Table 3:** **Variants from each category submitted to Affymetrix, classified by Affymetrix, and tiled on the final array**

	**SNP**	**mSNP**	**Indel**	**SNP-SNP**	**Indel-SNP**	**SNP-in-Insertion**	**F1D**	**Codon-based**
**Submitted candidates**	**159,721**	**1,940**	**12,801**	**7,764**	**2,937**	**4,921**	**17,518**	**28,646**
**Affymetrix classification**								
**Recommended (F)**	**81,792**	**380**		**3,598**	**433**	**487**	**3,185**	**5,327**
**Recommended (R)**	**7,576**	**359**		**2,441**	**541**	**1,465**	**3,262**	**4,876**
**Recommended (F & R)**	**23,556**	**470**				**296**	**1,013**	**12,928**
**Neutral (F)**	**815**	**170**	**1,035**	**464**	**125**	**355**	**1,356**	**939**
**Neutral (R)**	**834**	**147**	**3,520**	**589**	**107**	**216**	**1,225**	**886**
**Neutral (F & R)**	**2,298**	**476**	**4,999**			**41**	**109**	**1,774**
**Tiled on array**								
**No. of target sites**	**63,263**	**1,761**	**9,528**	**7,092**	**1,177**	**2,843**	**3,751**	**5,648**
**No of probesets**	**86,817**	**19,050**	**10,558**	**7,092**	**1,206**	**3,376**	**4,000**	**6,000**

Filtered candidates from each variant category were classified by the Affymetrix’ in silico design scores into ‘recommended’ and ‘neutral’ in the forward (F) strand, in the reverse (R) strand, and in both forward and reverse (F&R) strands. A ‘not-recommended’ class is not listed here as none of the candidates in this category were tiled on the array. Also listed are the number of markers or polymorphisms tiled on the array and the corresponding number of probe sets (one or more probes whose intensities are combined to interrogate a marker).

### Genotyping in octoploid strawberry

**Figure 3:**
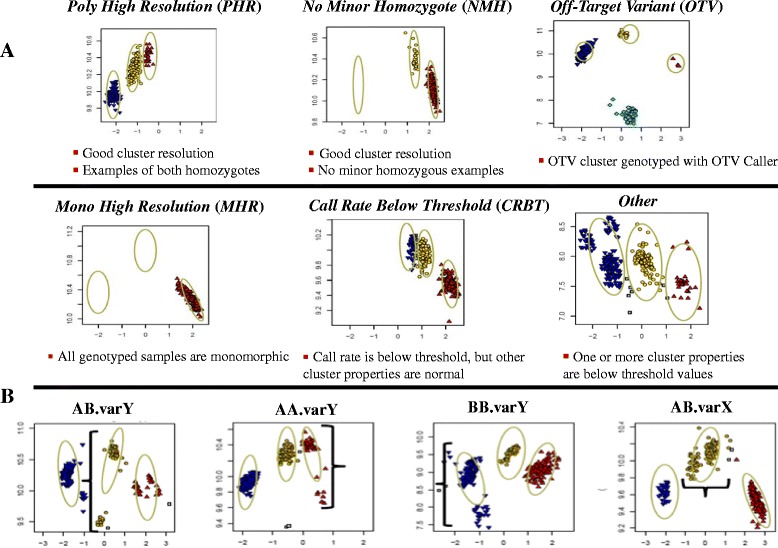
**Six SNP quality classes (A) and four variance filters (B) applied to the** ***PHR*** **genotype class. A**. Default SNP quality classes produced by the Axiom Best Practices Genotyping Workflow. **B**. An example cluster plot for a SNP identified with each of the four variance filters used including: AB.varY identified large heterozygous cluster variance in the Y dimension; AA.varY for homozygous (AA) variance in the Y dimension; BB.varY, for homozygous (BB) variance in the Y dimension; and AB.varX, for heterozygous variance in the X dimension.

DNA was isolated and quantified as described above for the octoploid strawberry validation set (Table 2). Up to 50 μL of DNA (≥20 ng/μL) from each sample was submitted to Affymetrix for genotyping. The Axiom assay was performed on four 96-sample Axiom arrays using the Affymetrix GeneTitan® system according to the procedure described by Affymetrix (http://media.affymetrix.com/support/downloads/manuals/axiom_2_assay_auto_workflow_user_guide.pdf). Next, cell intensity files (.CEL) generated by the GeneTitan instrument were converted to genotype calls using the Axiom Genotyping Algorithm version 1 (Axiom GT1) available through Affymetrix Power Tools or Genotyping Console™v4.1 software package. The procedure is documented by Affymetrix (http://media.affymetrix.com/support/downloads/manuals/axiom_genotyping_solution_analysis_guide.pdf). Executing the Axiom Best Practices Genotyping Workflow, SNPs sorted into six quality classes according to their clustering performance with respect to various quality-control measures (Figure 3A). These SNP classes were: (1) “*Poly High Resolution*” (*PHR*), which were polymorphic and passed all quality control (QC); (2) “*No Minor Homozygote*” (*NMH*), which passed all QC but only two clusters were observed; (3) “*Off-Target Variant*” (*OTV*), which had an additional low intensity cluster resulting from slight mismatches between the probe and the sequences for that group of individuals; (4) “*Mono High Resolution*” (*MHR*),which passed all QC but were monomorphic; (5) “*CallRate Below Threshold*” (*CRBT*), where genotype call rate was under 97%; and (6) “*Other*”, where the resultant SNP cluster pattern did not fall into any of the previous classes. SNPs that fell into the *OTV* class were further genotyped using *OTV* Caller, a statistical method developed by Affymetrix and included in the “SNPolisher” R package to identify samples that were homozygous for a null-allele.

### Cluster plot characterization

**Figure 4:**
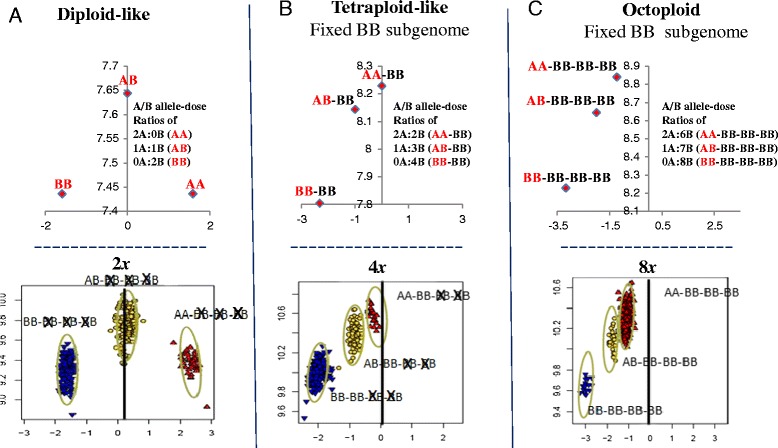
**Apparent polyploid levels based on comparing simulated to observed cluster locations.** Points for plots in upper row are the simulated cluster center locations for the given genotype where A = (#A_alleles * Intensity_per_A_allele) + Background_Intensity and B = (#B_alleles * Intensity_per_B_allele) + Background_Intensity. Intensity_per_A_allele = Intensity_per_B_allele = Background_Intensity = 100. The #A_alleles and #B_alleles are the counts of each allele in the given genotype. Points for plots in lower row are the observed contrast vs size values for each sample. Each column in 4A, 4B, and 4C is a SNP locus at a different polyploid level. An “*X”* is drawn over subgenome genotypes to indicate effective absence. A vertical bar is drawn at contrast = zero. **A**: The 2*x*/diploid-like cluster pattern. Alleles segregate in one subgenome and are effectively absent in the other three subgenomes. The AB cluster is centered ~ contrast = zero and the homozygous BB and AA genotype clusters have negative and positive contrast values, respectively, in the simulated and observed cluster patterns. **B**: The 4*x*/allo-tetraploid-like cluster pattern. Alleles segregate in one subgenome, are fixed in another subgenome, and are effectively absent in the other two subgenomes. One of the genotype clusters AABB, is centered near contrast = zero and the other two genotype clusters are offset to negative contrast values in both the simulated and observed cluster patterns, and correspond to one subgenome being fixed for the B-allele. **C**: The 8*x*/allo-octoploid-like pattern. Alleles segregate in one subgenome, and are fixed in at least two other subgenomes. Simulation is shown for allo-octoploid genotypes, where alleles segregate in one subgenome and three other subgenomes are present and fixed for the same allele. The pattern is the same as that for the 4*x* locus, except that all genotype clusters are offset to the positive (subgenomes are fixed for the A-allele) or negative (subgenomes are fixed for the B-allele, shown) contrast values.

The Homozygous Ratio Offset (HomRO) is a measure that allows automated discrimination between SNPs that display a diploid-like cluster (Figure 4A) as opposed to a polyploid-like cluster (Figure 4B-C). It measures the displacement from 0 contrast of the homozygous cluster closest to that value. From simulation results, SNPs in a diploid organism are expected to have a positive HomRO value and SNPs in a polyploid organism to have a negative (or near 0) HomRO value. Based on simulation, we used a HomRO value ≥0.3 to classify SNPs as clustering like a diploid, and a HomRO value <0.3 to classify SNPs clustering like a polyploid.

To examine the basis for diploid-like clustering as observed in ~30% of the di-allelic SNPs, we examined the relationship between clustering pattern and genomic read depth. First, we extracted read depth information from the BAM files and compiled corresponding lists of mean read depths for each of the 30,521 genes defined by the gene models for the *F. vesca* ‘Hawaii 4’ v1.0 reference genome [41]. The mean read depth of each gene was the average of the read depths at each nucleotide coordinate within that gene. The frequency (y axis) of each mean read depth category (x axis in unit increments from 0 to 150) was then plotted for each of the HD-20 octoploids.

### Genotyping in diploid strawberry

The Axiom assay was used initially to process all 27 diploid strawberry samples, and these samples were genotyped with the AxiomGT1 algorithm. All samples passed QC. As with the analysis of octoploid samples, the SNPolisher software was used to classify the SNPs into six quality classes according to their clustering performance. However, for these samples, the default diploid settings were used. Subsequently, the three *F. vesca* samples were excluded from the analysis, and genotype calling was repeated using only the 24 *F. iinumae* samples.

### Inheritance-based SNP validation

Genotyping accuracy of SNPs was estimated based on the proportion of SNPs that generated concordant genotype calls between parents and offspring using Microsoft® Excel, after excluding SNPs that had a missing genotype call in one or both parents in three biparentally derived populations: octoploids ‘Holiday’ × ‘Korona’ and its 79 F_1_ progeny (HK), and ‘Capitola’ × CF1116 and its 20 F_1_ progeny (CCF); and diploid (*F. iinumae*) J17 × J4, F1D, and its 21 F_2_ progeny (F2D).

### Linkage mapping of *PHR*, *NMH* and *OTV* SNPs in the ‘Holiday’ × ‘Korona’ population

SNPs were chosen that were polymorphic in at least one of the two parents of the mapping population, or, in case of *OTV* SNPs, that showed clear segregation in this mapping population despite monomorphic genotypes in both parents. *OTV* SNPs were hypothesized to be tri-allelic within a common subgenome, having a so-called null allele that did not show probe extension with labelled nucleotides in addition to the usual two alleles from the originally targeted polymorphism between the true nucleotides. Such null alleles are thought to represent deletions (indels) or to be due to additional SNP(s) at the probe site that hamper probe hybridization. Parental genotypes and progeny genotypes were deduced from their initial calls and from the marker segregation pattern. For instance, an AA × BB SNP that gave four cluster classes (AA, AB, BB, OTV) was reclassified as an A∅ × B∅ SNP that gave A∅, AB, B∅ and ∅∅ progeny genotypes.

To facilitate mapping efforts, markers were discarded that gave “NoCall” or that showed non-concordant genotype calls between parents and offspring for more than 5% of the progeny.

When non-concordant calls occurred for less than 5% of the progeny, such calls were assumed to be caused by inadequate genotype designation of some individual progeny samples rather than inadequate genotyping of the parents, and were converted to missing values. Integrated genetic linkage maps were constructed using the software JoinMap 4.1 [55] with the multipoint maximum likelihood mapping algorithm approach for cross pollinators [56] and the Haldane mapping function using all preset default settings for the calculation options. For linkage group 6D (LG6D), quality of genotype calls and of the presented maps was evaluated through a graphical genotyping approach [57] using Excel. Single data points causing double recombination events were checked by examination of the corresponding cluster plots.

Linkage groups were identified by integration of the SSR data of Van Dijk *et al.* [38] with the current SNP data. Once the SNP markers were grouped according to linkage groups, these SSR data were excluded from the generation of linkage maps in order to avoid issues of data consistency that may easily arise among markers from different experiments and genotyping platforms. Linkage groups were named according to Van Dijk *et al.* [38]: chromosome numbers followed the physical map of the diploid *F. vesca* ‘Hawaii 4’ [41] and subgenomes A to D were distinguished based on decreasing similarity to *F. vesca* genomic sequences as revealed by decreasing amplification efficiency of *F. vesca*-based SSR markers [38].

### Inheritance-based SNP validation in the *F. iinumae* F2D population

Genotype calls were obtained for the two crossing parents (J17 and J4), for F_1_ progeny plant F1D, and for 21 individuals of the derived F_2_ population (F2D). To be employed for mapping in the F2D population, SNPs that displayed segregation in the F2D population also had to be heterozygous in F1D, and the J17 and J4 parents could not share the same homozygous genotype. A genetic linkage map was constructed using the software JoinMap 4.1 [55] following the same procedures described above for ‘Holiday’ × ‘Korona’.

### Sequence-based SNP validation

Sequences of the shotgun genomic reads for each of the individuals in the octoploid, HD-20 subset, except for HolKor 2557 (Table 1, Additional file 1), were compared to genotype calls obtained only from the 9,186 di-allelic SNP markers classified as *PHR*. The genotype call rate was <97% in HolKor 2557 and genotype information was thus not available for analyses. Sequence-derived genotypes were only called when a minimum of 20 reads were present at the SNP site. For each SNP, the sequence-derived genotype was compared to the Axiom array-derived genotype when the latter was homozygous for either allele or if it was heterozygous; it was ignored when “NoCall” or null genotypes were obtained. To resolve homozygosity of SNPs with very few counts for the minor allele, a binomial test was performed where the null hypothesis was an expected minor allele frequency ≥1/8, (as expected from a heterozygous di-allelic subgenome-specific segregating SNP). An alpha value threshold of 0.05 was used to assign a homozygous (if p-value <0.05) or heterozygous genotype (if p-value ≥0.05) at a SNP position.

### Analyses of SNP data

Minor allele frequency (MAF) was calculated in 65 strawberry cultivars representing breeding selections and founders from Europe and across U.S. breeding programs in Florida, eastern U.S., California and the Pacific Northwest (Diversity set in Additional file 1) with an R script available in *plantbreeding* R software [58]. Distribution of SNPs was also drawn with *plantbreeding* R [58] by minor allele frequency according to physical location on the *F. vesca* ‘Hawaii 4’ v1.0 reference genome in 65 diverse strawberry accessions (Additional file 1).

## Results

### SNP discovery

After eliminating duplicate reads generated during the PCR enrichment of the libraries, mean depth of genome sequence coverage among the octoploids ranged from a low of 2× in *F. virginiana* CFRA 1992 (BC6) to a high of 48.7× in ‘Winter Dawn’ (Table 1). Upon visual examination of read alignments using Integrated Genome Viewer (IGV) [59], numerous instances of misalignment were observed at the ends of reads, especially in the presence of repetitive motifs. Implementation of GATK [54] for correction of local misalignment was effective in correcting such misalignments, and resulted in the elimination of numerous artifactual variants while increasing the read counts of some actual variants.

From the 20 octoploid VCF files, a total of 36,140,217 unique variants distributed over 10,619,615 coordinate sites in the ‘Hawaii 4’ (v 1.1) reference genome were discovered. The number of variants exceeded the number of sites because multiple variants could exist at any given site. As reported in the VCF files, these variants were of three basic types relative to the reference: *snp* (SNP), *ins* (insertion), and *del* (deletion). Beginning with the input information of the 36,140,127 variants and their respective genomic coordinates (sites), outputs were generated from the various filter pipelines, as described below and in Table 3.

### Di-allelic SNP pipeline (Additional file 3)

A total of 159,721 di-allelic SNP candidate sites was identified, and submitted to Affymetrix. Of these, 89,368 were classified as either recommended for only the forward or reverse strand (81,792 or 7,576, respectively) or for both strands (23,556) (Table 3). In total, 63,263 (32,135 tiled on forward strand; 7,574 tiled on reverse strand; and 23,554 tiled on both strands) di-allelic SNPs were chosen for inclusion on the array (Table 3). Given that these candidate SNPs passed the A/T-G/C exclusion, each could be genotyped using a single probe. However, for 23,554 of the 23,556 BothSafe candidates, we elected to “tile” (i.e., probe) both forward and reverse strands, necessitating the use of two probes. Tiling probe-sets on both strands as opposed to one strand substantially reduced the proportion of markers that did not work, as estimated from the lower proportion of SNPs classified into “*other*” (2.5% vs 13.6%), and “*CRBT*” (4.1% versus 8.2%), as well as the higher proportion of markers that were converted into *PHR* (18.8% vs 12.0%) (Additional file 11).

### Multi-allelic SNP pipeline (Additional file 4)

A total of 1,940 potentially suitable mSNP sites comprising 1,878 tri-allelic and 62 tetra-allelic sites were identified for submission to Affymetrix. A total of 1,414 sites were suitable for tiling on both strands, each requiring eight distinct probe sequences, while 347 were chosen for tiling on one strand, each requiring four distinct probe sequences. Thus, 1,761 mSNP sites were represented on the array (Table 3), using a total of 12,700 distinct probe sequences for a total of 19,050 probesets (Additional file 10).

### Di-allelic indel pipeline (Additional file 5)

A total of 12,801 indel sites were discovered. Of these, 9,554 passed the Affymetrix quality criteria, enabling tiling on one or both strands. In total, 9,528 indel sites were represented on the array (Table 3). Probes were designed on both strands for 1,030 of these sites, and 3,396 sites contained A/T or G/C sites that required two allele-specific probes per site, for a total of 13,954 distinct probe sequences interrogated by 10,558 probesets (Table 3, Additional file 10).

### HaploSNP pipelines (Additional files 6, 7 and 8)

Variants qualifying as marker SNP candidates in the haploSNP pipelines were required to be present in at least two germplasm panel members and absent in at least two others, upon which basis they were considered likely to be true marker SNPs and not HSVs. In contrast, the “critical” HSV candidates were required to be present in all panel members, upon which basis they were considered likely to be true HSVs and not marker variants. For the SNP-SNP category, the pipeline produced 7,764 candidate sites, of which 7,092 were included in the array using 7,092 probes (Table 3). For the Indel-SNP category, the pipeline produced 2,937 candidate sites, of which 1,177 were included in the array using 1,206 probes. For the SNP-in-Insertion category, the pipeline produced a total of 4,921 candidate sites, of which 2,843 were included in the array using 3,376 probes (Table 3, Additional file 10).

### Diploid (*F. iinumae* F1D) SNP discovery (Additional file 9)

Mean read depth in the *F. iinumae* VCF file was 36× (Table 1). A total of 8,373,559 variants were identified in comparison to the ‘Hawaii 4’ reference genome, and 17,518 SNPs passed the filtering criteria. Of the 3,751 included on the array (Table 3), 3,501 were tiled on a single strand, and 249 were tiled on both strands (Additional file 10). The F1D probe sites were chosen to be widely distributed across the seven linkage groups defined by the ‘Hawaii 4’ genome and to exclude duplication of octoploid-derived and codon-based probes sites.

### Codon-based SNPs

A total of 28,646 proline codons were identified at sites that qualified as BothSafe in relation to the GDP. Of the 5,648 included on the array, 5,296 were tiled on a single strand and 352 were tiled on both strands. The 5,648 marker sites (Table 3, Additional file 10) were chosen to be widely distributed across the seven linkage groups defined by the ‘Hawaii 4’ genome, and to exclude duplication of discovered (octoploid and F1D) SNP sites.

### SNP genotyping in octoploid strawberry

The goal of the Axiom Best Practices Genotyping Workflow and SNP quality control procedures is to identify SNPs whose microarray intensities form three well resolved genotyping clusters for two segregating alleles from a single locus in a single subgenome. The genotyping algorithm (AxiomGT1) automatically assigns genotypes to the samples in such clusters (Figure 3A, *PHR*). The position of these clusters is expected to vary with the number of non-segregating subgenomes that are targeted in addition to the segregating subgenome. This behavior is consistent with the differing reduction of the polyploid levels at these loci (Figure 4). However, the genotyping algorithm was designed to dynamically adapt to such variation in cluster locations.

**Figure 5:**
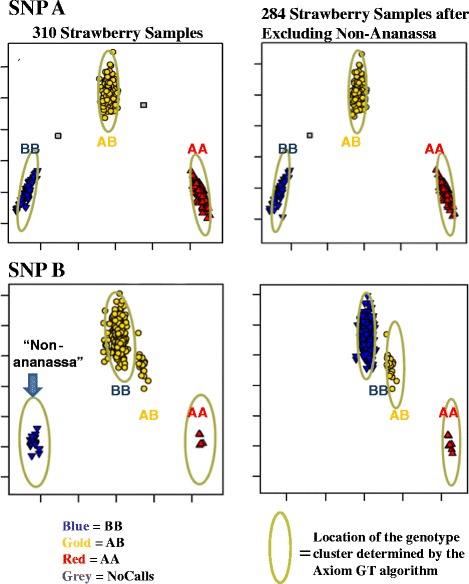
**Effect of excluding 51 “non-ananassa” octoploid accessions from genotyping co-cluster runs for two SNPs.** The cluster plots in the left column are for 310 strawberry samples. The cluster plots in the right column are for 284 strawberry samples, excluding the non-ananassa samples (*F. chiloensis* and *F. virginiana*). SNP A is a robust diploid-like SNP site that produces only three genotype clusters in the presence as well in the absence of the wild progenitor samples, and thus is genotyped correctly in both circumstances. SNP B is a more challenging SNP site at which the genomes of the divergent non-ananassa form a 4th genotype cluster (arrow). This 4th cluster causes the samples in the BB homozygous genotype cluster to be incorrectly called as heterozygous (colored gold). When the non-ananassa samples are excluded (bottom right), the software correctly calls the samples in the homozygous BB cluster as “BB” (colored blue).

The first attempt at genotyping the polyploid samples included the 306 *F.* ×*ananassa* samples and 51 octoploid “non-ananassa” accessions (Table 2, Additional file 1). For some SNPs, adequate genotyping was hampered due to the inclusion of different octoploid species, which caused samples to form more than three intensity clusters (Figure 5). SNPs with such complex cluster groups resulted in a higher error rate of automated assignments of genotypes to clusters. In addition, samples whose intensities fell between the three prototypical clusters were more likely to have miscalled genotypes. While procedures and filter thresholds proposed by Affymetrix adequately identified SNPs with complex cluster patterns in diploid species, they needed to be extended for use with allo-polyploid species. Therefore, a second attempt was performed using advanced approaches to identify such SNPs and samples and filter them appropriately.

Three steps were taken to further analyze SNPs whose intensities formed only three well resolved genotype clusters typified by the *PHR* class, as these SNP were thought to have the best prospects for application across highly diverse germplasm. These steps were also aimed at excluding samples whose intensities fell between the prototypical clusters.

#### Step 1 Only co-cluster *F.* ×*ananassa* accessions

As compared with the *F.* ×*ananassa* samples, the 51 octoploid “non-ananassa” samples tended to cluster more often in their own intensity space, and so their inclusion produced more than three intensity clusters for some SNPs (Figure 5). Therefore, only the 306 *F.* ×*ananassa* samples were analyzed as a batch with the Best Practices Genotyping Workflow. Two hundred and eighty four of these samples passed all sample QC filters (Additional file 1) and were co-clustered by the AxiomGT1 algorithm to produce genotype calls (AA, AB, BB, and No Calls).

#### Step 2 Execute axiom best practices genotyping workflow

The Axiom Best Practices Genotyping Workflow sorted SNPs into the six quality classes as described in the Methods (Figure 3A, Table 4). Three of the six classes generated accurate genotypes and deserved further validation: *PHR*, *NMH* and *OTV* classes. The remaining three SNP classes were either not informative (*MHR*) or had intensity clusters that were too complex to fit a simple three-state genotype model (*Other*, *CRBT*) (Figure 3A).

**Table 4:** **Number of SNPs in six classes after applying reproducibility, variance and nMinorHom filters**

**SNP class**	**No. of SNPs**	**No. of SNPs in category**							
		**SNP**	**mSNP**	**Indel**	**SNP-SNP**	**Indel-SNP**	**SNP-in-Insertion**	**F1D**	**Codon-based**
***MHR***	18,958	9,913	191	2,458	737	286	1,114	1,864	2,395
***CRBT***	5,876	4,248	0	542	761	96	144	44	41
***Other***	9,755	6,014	0	1,214	1,387	196	428	150	366
***NMH***	36,088	25,782	1,050	3,365	979	298	843	1,498	2,273
***OTV***	1,030	415	1	106	126	21	11	38	312
***PHR***	23,355	16,891	519	1,843	3,101	280	303	157	261
Reproducibility	3,263	2,568	129	200	182	17	39	55	73
AAvarianceY	491	339	10	38	82	8	8	1	5
ABvarianceX	1,775	1,095	11	221	328	32	32	18	38
ABvarianceY	3,212	2,367	54	279	384	51	34	14	29
BBvarianceY	394	183	6	43	144	7	5	2	4
nMinorHom	1,611	1,153	87	176	53	17	31	33	61
**Filtered** ***PHR***	12,609	9,186	222	886	1,928	148	154	34	51
**No of markers on array**	95,063	63,263	1,761	9,528	7,092	1,177	2,843	3,751	5,648

These SNP classes were: *MHR* (*Mono High Resolution*), which passed all QC but were monomorphic; *CRBT* (*CallRate Below Threshold*), where genotype call rate was under 97%; *Other*, where the resultant SNP cluster pattern did not fall into any of the other classes; *NMH* (*No Minor Homozygote*), which passed all QC but only two clusters were observed; *OTV* (*Off-Target Variant*), which had an additional low intensity cluster resulting from mismatches between the probe and the sequences for that group of individuals; and *PHR* (*Poly High Resolution*) which were polymorphic and passed all QC. *PHR* SNPs were subjected to additional filters (reproducibility, variance and nMinorHom).

While *PHR* SNPs indicated high quality because they had three well-distinguished clusters, many SNPs in the *NMH* class were also of high quality. Lack of one of the classes may have a clear genetic reason, such as limited representation of genetic variation within the examined germplasm, low frequency of the minor allele for the SNP, or SNP localization in a recently introgressed region or linked to a gametophytic incompatibility locus or a recessive gene with deleterious effects. The *OTV* class contained SNPs for which some individuals fell in a subcluster with lower hybridization signal and that can be genotyped as homozygous for a so-called null allele. *OTV* Caller was used to further genotype 1,030 *OTV* SNPs, which resulted in 831 in the *OTV*-*PHR* class, one as *NMH*, 167 SNPs that were classified as *CRBT*, and 31 in *Other*. For the *OTV-PHR* SNPs, homozygous null genotypes (∅∅) were assigned to the individuals in the low hybridization zone or OTV cluster.

Even with the exclusion of the 51 octoploid “non-ananassa” samples, strawberry samples formed complex cluster groups for many SNPs, and not all such SNPs were excluded from the default *PHR* class. Therefore, the 23.4 K default *PHR* SNPs were subjected to six additional filters developed or optimized for the strawberry data (Table 4). These filters included:

1. A reproducibility filter that required 100% reproducibility of technical replicates, thus removing SNPs with potentially higher error rates due to various sources.
2. Four *Variance* filters that specifically targeted SNPs with more than three genotype clusters (Figure 3B).
3. An *nMinorHom* filter that excluded SNPs with less than n samples of called minor homozygous genotypes. A threshold of 3 was used, which excluded SNPs for which less than three samples clustered in their own intensity space. Such small sample sets may be true clusters but may also be an additional cluster causing the true AA, AB, and BB genotype clusters to be fused and miscalled.

This advanced filtering decreased the number of SNPs in the *PHR* class from 23.4 K to 12.6 K (Table 4) and greatly increased the quality of the SNPs based on visual examination.

#### Step 3. Increase the stringency of sample calling

Steps 1 and 2 are effective in identifying SNPs with three potentially well resolved genotype clusters. However, the error rate, due to individual samples falling between clusters or being located at cluster junctions, is likely to be higher at higher ploidy levels (as the AB cluster approaches one of the homozygous clusters) (Figure 3). To address this problem, the stringency of sample calling was increased so that in a final step, such samples in the *PHR* SNPs were set to “NoCall” (Additional file 12). The AxiomGT1 algorithm uses a confidence score of 1 – p(X), where X is the assigned genotype call. For strawberry, the default setting of 0.15 was replaced by 0.01 for the genotype calls used in linkage mapping and data analyses.

### Genotyping success

**Figure 6:**
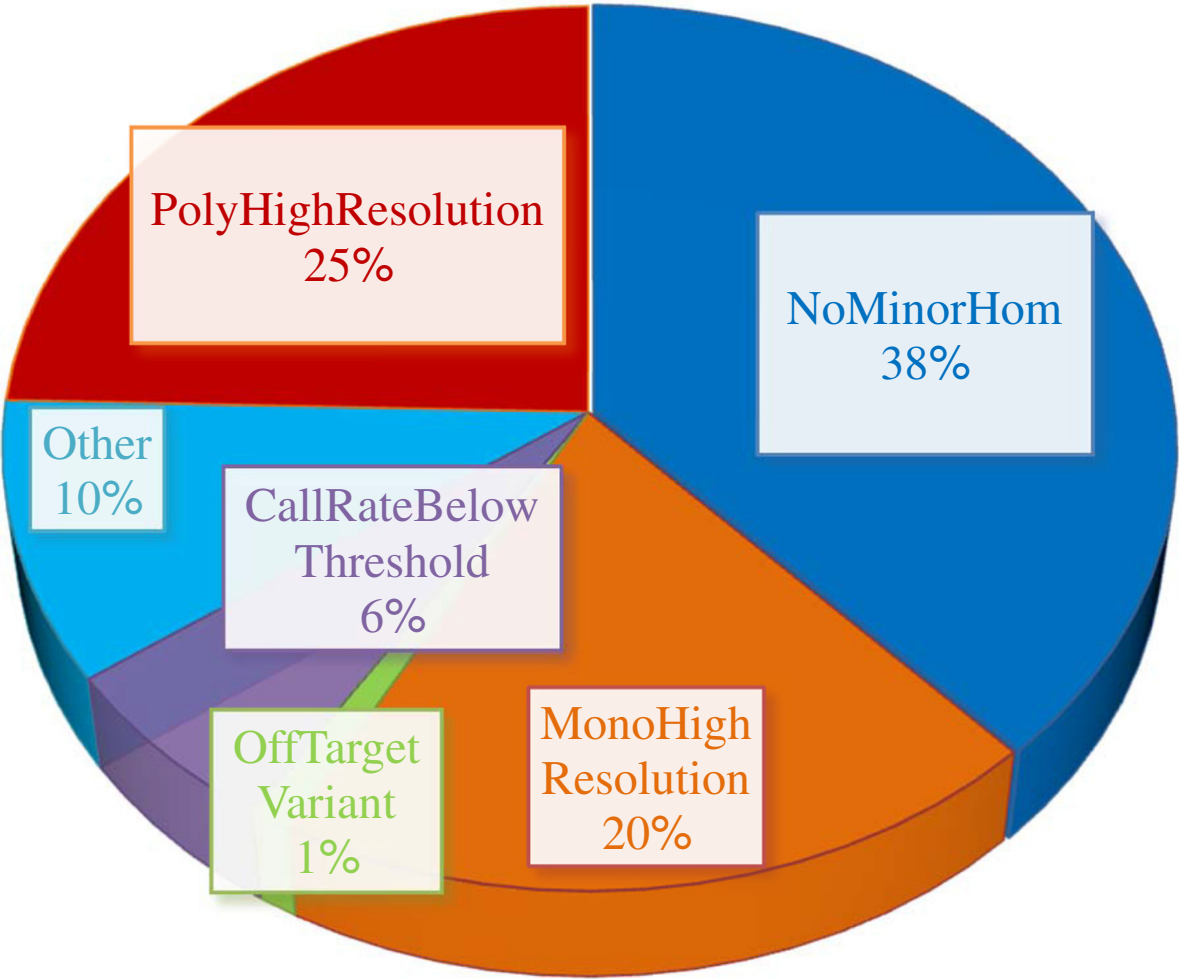
**Proportion of 90K array markers classified into each of the six classes by “SNPolisher”.**

**Figure 7:**
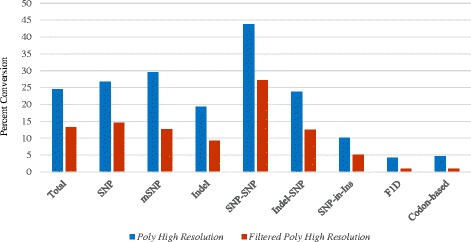
**Conversion rate of each SNP category into the** ***PHR*** **and filtered** ***PHR*** **class of markers.** SNP categories consisted of: standard di-allelic SNPs; multi-allelic SNPs (mSNPs); indels; reduced ploidy haploSNPs, including SNP-SNP, Indel-SNP, and SNP-in-Insertion (SNP-in-Ins); *F. iinumae* F1D SNPs; and speculative codon-based SNPs.

The conversion rates of SNPs into the *PHR* and filtered *PHR* classes was considered a measure of genotyping success. Prior to advanced filtering, the *PHR*, *NMH*, and *OTV* classes comprised 25%, 38%, and 1%, respectively, of array SNPs (Figure 6). After implementing the additional filters described above, 13% of the 95 K SNPs interrogated on the array fell into the *PHR* class, resulting in 12,609 “filtered” *PHR* SNPs (Table 4, Figure 7, Additional file 10). A large variation was observed in the proportion of SNPs that fell into this class based on the SNP category and haploSNP marker type (Figure 7). Conversion into filtered *PHR* SNPs was 14.5% for the standard di-allelic SNPs, and averaged 9.3% for indels, and 20% for haploSNPs. Conversion rate was highest in the SNP-SNP approach (27%) and lowest in the SNP-in-Insertion category (5.4%) (Figure 7).

Conversion rate of F1D SNPs and codon-based SNPs into filtered *PHR* SNPs was <1% (Figure 7) in the octoploid *F.* ×*ananassa* validation set (Table 2, Additional file 1). In comparison, an 84% conversion rate from candidate to *PHR* class was achieved for the 3,751 F1D arrayed SNPs in relation to the diploid *F. iinumae* validation set.

### Cluster characteristics

**Figure 8:**
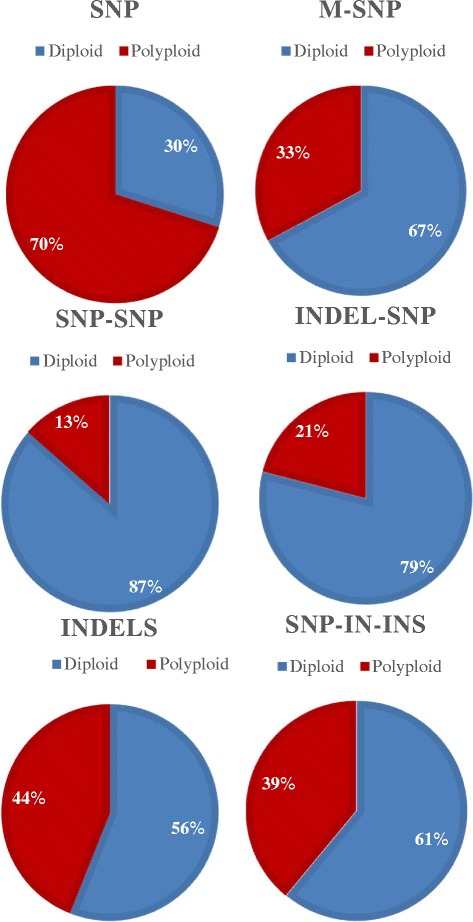
**Proportion of diploid- and polyploid-clustering SNPs in the SNP categories designed from the octoploid GDP.** Marker categories include di-allelic SNPs, multi-allelic SNPs (mSNPs), indels, and the three haploSNP categories of SNP-SNP, Indel-SNP, and SNP-in-Insertion.

The HomRO parameter indicated that, in the di-allelic SNPs, 30% of the 12,609 filtered *PHR* SNPs generated diploid-like clusters while 70% had polyploid-like clusters (Figure 8, Additional file 10). In the mSNPs, indels and haploSNPs, 56-87% of the SNPs generated diploid-like clusters while 13-44% had polyploid-like clusters (Additional file 10). SNP-SNPs generated the largest proportion of diploid-like clusters at 87% (Additional file 10). The contrasting diploid-like clustering percentages for di-allelic SNPs versus the SNP-SNPs clearly indicates that the intended ploidy reduction effect was achieved by the haploSNP strategy.

**Figure 9:**
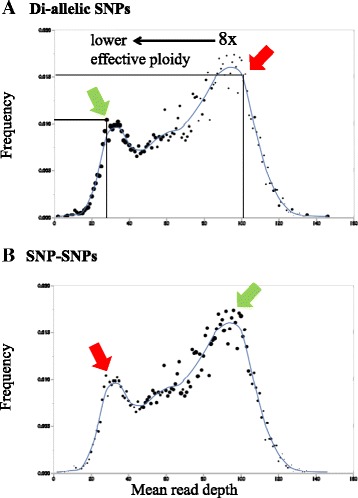
**Relationship of diploid clustering to mean genic read depth in** ***F.*** **×** ***ananassa*** **‘Winter Dawn’, displayed for** ***PHR*** **di-allelic SNPs (A) and for** ***PHR*** **SNP-SNPs (B).** Black dot positions, which are identical in panels **A** and **B**, represent the mean read depth category (X axis), and the relative frequency of this read depth category (Y axis). The size of each dot is directly proportional to the fraction of di-allelic SNP **(A)** or SNP-SNP **(B)** markers that displayed diploid-like clustering. For example, the green arrow in **(A)** points to a large black dot with mean read depth category of approximately 28× (X axis) and a category frequency of approximately 0.01 (Y axis). The red arrow in **(A)** points to a small black dot corresponding to a mean read depth category of about 100× and a category frequency of about 0.015. The larger dot sizes (green arrows) occur in **(A)** in regions of comparatively low read depth, and in **(B)** in regions of comparatively high read depth. Conversely, smaller dot sizes occur in **(A)** in regions of comparatively high read depth, and in **(B)** in regions of comparatively low read depth (red arrows).

As exemplified by the plot for *F.* ×*ananassa* ‘Winter Dawn’ (Figure 9), the read depth distributions of SNPs were distinctly bi-modal and sometimes tri-modal, indicating an underlying discontinuous distribution as would be expected if the genome was partitioned into regions that were effectively diploid, tetraploid, hexaploid, or octoploid, the latter being the predominant component (Figure 9: highest peak in distribution). When frequencies of *PHR* markers that exhibited diploid clustering in each read depth category were computed and depicted graphically for di-allelic SNPs (Figure 9A) and for SNP-SNPs (Figure 9B), diploid clustering for the di-allelic SNPs was most prevalent at marker sites that displayed reduced read depth, as would be expected if elevated diploid clustering was related to localized biological (i.e., actual) ploidy reduction. In contrast, diploid clustering for the SNP-SNPs was more prevalent in the zones of higher read depth, and particularly at the presumed octoploid level, indicating that the diploid clustering resulted from the effectiveness of the technical ploidy reduction strategy and not from underlying biological ploidy reduction.

### Inheritance-based SNP validation: Parent–child concordance

Genotyping accuracy was estimated for the 12,609 filtered *PHR* SNPs using the proportion of SNPs that generated concordant genotypes between parents and offspring in two octoploid biparental populations: ‘Holiday’ × ‘Korona’ and its 79 progeny (HK), and ‘Capitola’ × CF1116 and its 20 progeny (CCF). In the HK progeny, of the 12,089 filtered *PHR* SNPs that had genotype calls in both parents, 10,961 or 91% of the SNPs had concordant genotypes while 1,128 SNPs or 9.3% had at least one progeny with a genotype call that was not consistent with its pedigree. In the CCF progeny, 9,471 of 11,019 (86%) had genotypes that were consistent with declared pedigree while 1,548 (14%) had at least one progeny with genotype inconsistencies. Additional filtering for robust performing SNPs will leave sufficient numbers of SNPs to allow for integrated QTL analyses across multiple pedigreed families by the Pedigree Based Analyses approach [60].

In diploid *F. iinumae*, genotyping accuracy was evaluated in two ways. For the 3,171 *PHR* F1D SNPs, 3,023 (96%) were concordant between the two parents (J17 and J4) and F1D. In all 148 instances of non-concordance, F1D was genotyped as heterozygous while J17 and J4 had identical homozygous genotypes. All of the 3,023 *PHR* F2D SNPs that were parentally concordant and genotyped as heterozygous in F1D also displayed segregation in the F2D population.

### Inheritance-based SNP validation: performance in mapping

Of the 12,609 filtered *PHR* markers, 4,005 (32%) were monomorphic for the HK-family, being homozygous in both parents. Also, 520 SNPs (4%) had a missing genotype call for at least one parent of which 126 were segregating in HK. These 520 SNPs were discarded from the current mapping effort because absence of parental genotypes was assumed to indicate non-robust genotyping performance and therefore a potential source of major mapping problems. The remaining 8,084 (64%) filtered *PHR* SNPs (Additional file 10) were polymorphic in at least one parent and were further processed. Of these, 1,140 (14%) were discarded because more than 5% of the progeny showed “NoCall” and/or marker genotypes that were non-concordant with the parents. During the map construction process with the remaining 6,944 markers, another 284 markers were discarded because of poor integration, giving rise to considerable stress or mapping at large distances from any other marker, or because they mapped to a small linkage group that could not be assigned to any of the 28 chromosome pairs. In this first mapping effort, of the initial 8,084 HK-polymorphic *PHR* SNPs, 6,696 (83%) were successfully mapped across all 28 chromosome pairs. The successful incorporation of a segregating SNP or indel marker into the linkage map was considered to provide conclusive evidence that the respective variant was not an HSV or PSV.

**Figure 10:**
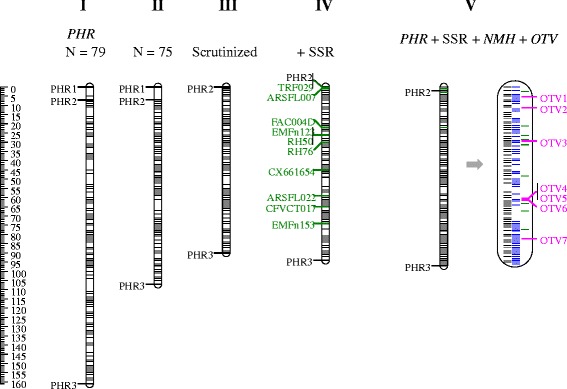
**Five SNP linkage maps for linkage group 6D.** Maps were derived from successive steps in map construction: I) 413 *PHR* SNPs on the full family (n = 79); II) using 75 progeny (four individuals removed due to poor performance based on graphical genotyping results or because they were duplicates; III) after scrutinizing genotype calls, some data points were replaced with missing values thus removing singletons and a pair; IV) adding 10 previously mapped SSR loci [38]; and V) a full data set totaling 10 SSRs and 667 SNPs consisting of 413 *PHR*, 247 *NMH* and 7 *OTV* SNPs. Black, blue and pink locus lines indicate *PHR*, *NMH* and *OTV* SNPs respectively, and green lines indicate SSR loci. The outer *PHR* SNPs of the maps are highlighted. *PHR* markers 1 to 3 refer to AX-89840764, AX-89799050 and AX-89799050 respectively. *OTV* markers 1 to 7 refer to the SNP (of the cross) AX-89814809 (∅∅ × A∅), AX-89869370 (∅∅ × A∅), AX-89866711 (A∅ × ∅∅), AX-89871559 (A∅ × ∅∅), AX-89896961 (AB × BB), AX-89897092 (AB × BB), and AX-89808404 (A∅ × B∅), respectively.

Marker performance and map quality were further examined by a more in-depth analysis of LG6D through graphical genotyping of the single parent maps. Two offspring showed a high number of singletons (isolated double recombinants) that could not be reduced by alternative maps of at least equal quality. Two further offspring were represented twice (identical SNP profiles). These results from the four individuals indicated errors in labeling of samples after the SSR genotyping of HK [38]. Exclusion of data from these offspring resulted in a reduction in map size from 163 cM to 106 cM (Figure 10I and II). Graphical genotyping showed the ‘Holiday’ map to still be of poor quality with several blocks of multiple SNPs that had double recombination in multiple offspring (Additional file 13). The ‘Korona’ map had a block of SNPs with high degree of double recombination on LG6D. For both maps, these double recombinant regions could be resolved by manual adjustment of marker order, leaving five singletons for ‘Holiday’ (of which three were for the same marker) (Additional file 13) and one pair and three singletons for ‘Korona’. Genotype calling of these singletons and pair was validated through inspection of the relevant cluster plots. Due to their major negative impact on obtaining good quality high-density SNP maps, these data points were rescored as missing. The resulting integrated linkage map had a length of 90 cM (Figure 10III), and the JoinMap-derived single parental maps no longer contained suspicious double recombinations. We therefore concluded that the presence of even very few incongruent data points (<0.05%) had a major impact on the quality of a high marker density genetic linkage map. Once these were removed, previous marker data for ten SSR loci [38] could be easily integrated with the current SNP data (Figure 10IV). Graphical genotyping plots demonstrated lack of double recombination and the map size remained stable (Figure 10IV).

**Figure 11:**
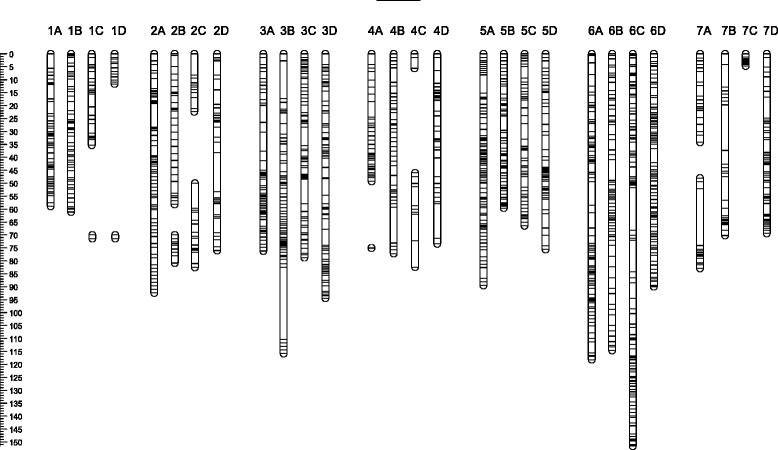
**Integrated** ***PHR*** **-SNP linkage map of allo-octoploid strawberry using the ‘Holiday’ × ‘Korona’ family (n = 75).** A total of 6,593 markers were placed on this map. Linkage groups are named according to Van Dijk *et al*. [38]. Large gaps mostly coincide with homozygous regions as revealed by SSR-haplotype profiles [38]).

Removing the data from the four above-mentioned offspring and another 102 SNPs that were easily identified as causing major problems in mapping gave 6,594 mapped *PHR* SNPs (Additional file 10), which resulted in a genetic map of 2,050 cM, accounting for 82% of the initial 8,084 *PHR* SNP that were polymorphic in HK. The genetic length of the individual linkage groups varied greatly (Figure 11). The mapped SNPs showed an uneven distribution across the subgenomes as most markers were from subgenome A, followed by B (Figure 11, Table 5). Overall, subgenomes C and D had the least number of markers, except in LG6C & 6D (Table 5). This general pattern was in accordance with commonalities in the approaches for subgenome assignment and that for designing SNPs, as both used *F. vesca* genomic sequences as a reference. Subgenomes A to D were distinguished by decreasing similarity to the *F. vesca* genomic sequences as revealed by decreasing amplification efficiency of *F. vesca*-based SSR markers [38]. Design of the array SNP markers included a step of aligning re-sequencing data to the published *F. vesca* genome sequence. Consequently, subgenomes C and D were expected to have the largest sequence divergence to *F. vesca* based on SSR data, and were thus expected to have a lower proportion of their re-sequenced fragments aligned to the reference genome sequence, which would lead to reduced representation of corresponding SNP markers.

**Table 5:** **Number of SNPs mapped in 'Holiday' x 'Korona' to the four subgenomes of** ***F.*** **×** ***ananassa***

**LG**	**Subgenome**			
	**A**	**B**	**C**	**D**
**1**	398	181	93	50
**2**	325	151	119	156
**3**	392	293	241	208
**4**	204	242	61	151
**5**	548	292	152	237
**6**	537	228	266	413
**7**	202	191	93	170
**Total**	**2606**	**1578**	**1025**	**1385**

This general tendency is expected to occur when genetically diverse germplasm is investigated. For a specific family, such a general tendency might be counteracted by differences in homozygosity between the subgenomes. For the HK SNP linkage maps, two chromosomes followed the expected general trend (Chromosomes 1 and 3, Table 5). Relatively high levels of homozygosity may explain the relatively low number of SNP markers for LG4C and LG7C [38]. Also, deviations from the expected general pattern might be due to differences between the SSR-estimated and true sequence similarities. Such deviations might have occurred for LGs 4A, 4B, 5C, 5D, 6C and 6D for which only a low number of *F. vesca*-derived SSR loci were analyzed and for which differences in amplification efficiency were minor.

The 36 K *NMH* and 1 K *OTV* SNPs provided an additional reservoir of thousands of potentially useful markers. Of these, 10,419 *NMH* SNPs (29% of *NMH* SNPs on the array) and 353 *OTV* SNPs (34% of *OTV* SNPs on the array) were polymorphic in HK, of which 8,068 (77%) and 331 (94%), respectively, met quality criteria for inclusion in the mapping process. *NMH* and *OTV* SNPs integrated well into the *PHR* maps, as demonstrated for LG6D (Figure 10V). Of the initial 263*NMH* and 8 *OTV* SNP evaluated for LG6D mapping, 16 (15*NMH* and one *OTV*) were removed due to their poor performance in JoinMap. The resulting map was 113 cM long and was free of double recombinants, with just one pair and seven singletons for ‘Holiday’ (of which three were for the same marker) and five singletons for ‘Korona’. The pair and singletons were caused by a single *NMH* SNP, but one that was related to an SSR marker. Removal of the marker with multiple singletons and rescoring the data for the other singletons as missing resulted in a map of 97 cM. The addition of the 247 *NMH* and 7 *OTV* markers (Additional file 14) thus resulted in good integration into the *PHR* framework map of LG6D (Figure 10) and a 62% increase in marker density to the initial *PHR* map of 413 SNPs for this linkage group (Table 5). The actual chromosomal fragment that was represented by markers was thus hardly affected by inclusion of *NMH* and *OTV* SNPs (Figure 10V). The respective increase and decrease in the corresponding genetic maps thus indicates the occurrence of occasional genotyping errors in a large series of overall well-performing markers.

The presence of some genotyping problems was revealed during the mapping process. Of the 353 HK-polymorphic *OTV* SNPs, a large group of 121 SNPs showed a similar pattern of non-concordance for parent-progeny genotypes that could not be solely explained by the introduction of null alleles. For the HK progeny, these *OTV* SNPs showed a clear 1:1 segregation for the AA and BB clusters whereas the parents were genotyped as homozygous AA and BB or BB and AA. The 1:1 segregation indicated segregation from just a single parent. To examine their performance in mapping, these SNP data were scored as two alternatives, either as a maternal or as a paternal marker. The alternative that integrated well into the *PHR* framework map was maintained. In this way, three such markers could be well integrated into LG6D (See OTV-4, −5, and −6 SNPs of Figure 10). Examination of their cluster plots identified this *OTV* subset to be of type *NMH* whereby the heterozygous genotype was erroneously classified as homozygous (Additional file 15). This analysis allowed for correctly identifying the heterozygous parent. Two of the three markers were of type AB × BB (*OTV*5 & *OTV-*6) and one of type A∅ × ∅∅ (*OTV*-4) Additional file 15).

**Figure 12:**
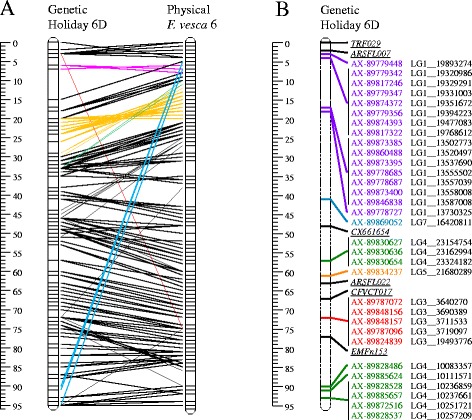
**Relationship between the genetic map of LG6D of the allo-octoploid cultivar Holiday and that of the diploid cultivar** ***F. vesca*** **‘Hawaii 4’.** Panels **A** and **B** present SNPs and SSRs that physically came from LG6 or other LGs, respectively. The rulers indicate genetic distances in cM and physical distances in bases/ 403,743 (thus unifying scales). Different colours highlight cases of large discrepancies (Panel **A**), or indicate different physical LGs (Panel **B**). Panel **A**: Red & blue, proximal markers becoming distal and vice versa; Green, medium shift in position; Pink and orange, opposite order of multiple SNPs for a small and large chromosomal segment respectively.

Initial comparisons between physical and genetic maps demonstrated the prospects of the SNP array for further improvement of the physical map, including re-orientation of contigs and scaffolds, re-allocations to different regions within the assigned LG, and even re-assignment to other LGs (Figure 12, Additional file 14).

### Validation of F1D SNPs by mapping in *F. iinumae*

Of the 3,023 F1D SNPs that were classified as *PHR* and that were parentally concordant, all but 14 (0.46%) were able to be incorporated into a linkage map of the expected seven linkage groups based upon segregation in the 21-member F2D population. Also mapped in the F2D population were 177 octoploid-derived markers (not limited to the *PHR* category) and seven codon-based markers. The resulting *F. iinumae* linkage map will be presented elsewhere.

### Sequence-based validation

**Figure 13:**
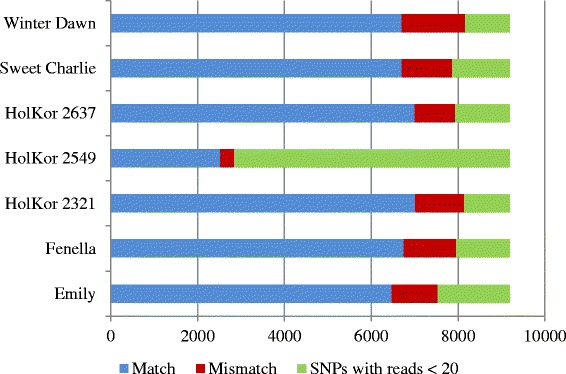
**Matching and non-matching HD-20 genotypes obtained by comparing sequence-derived to array-obtained genotypes.** Genotype data was not available for HolKor 2557 as the genotype calls were < 97%. No genotype comparison was possible when read depth at the variant site was less than 20 (green bars).

For seven of the eight HD-20 filtration panel members (excluding HolKor 2557 for which genotype data was not available), comparisons were made between sequence-derived genotype calls and those of 9,186 di-allelic *PHR* SNPs. In total, 64,302 comparisons were attempted (9,186 SNPs x 7 individuals). A comparison could not be made if the SNP had “Nocall” in the detection panel for a given individual, fewer than 20 sequencing reads were present at the SNP location, or the SNP had more than two alleles at the SNP location. At least one comparison was possible for all but 22 of the 9,186 SNPs. These SNPs failed to meet the requirements for all seven individuals of the HD-20 panel. In total, 50,457 comparisons were possible, and of these, 43,153 (85.5%) had the same genotype calls from both the Axiom array and sequencing data. Of the 7,304 (14.5% of total) comparisons that did not have matching genotype calls, 6,425 (87.9%) were heterozygous based on sequencing and had homozygous Axiom array genotype calls, likely caused by sequencing or genotyping error. Another 839 (11.5%) appeared homozygous based on sequencing, and were heterozygous based on array genotyping, possibly due to absence of allele representation of alternate allele in sequence or presence of signal from paralogous sequences; and 40 (0.5%) had alternative homozygous genotypes. The number of comparisons for an individual appeared to be highly correlated with sequence read coverage. A smaller number of SNPs were compared in the HD-20 member with the lowest genome sequence coverage (HolKor 2549), as opposed to the remaining six HD-20 with higher genome coverage (Table 1, Figure 13).

### Codon-based approach

Of the 5,648 codon-based markers placed on the array, 51 (0.9%) were classified into the filtered *PHR* category, and none of these achieved incorporation into the ‘Holiday’ x ‘Korona’ linkage map. As previously noted, seven of 5,648 (0.12%) codon-based markers were incorporated into the *F. iinumae* map.

### SNP polymorphism in the cultivated strawberry

**Figure 14:**
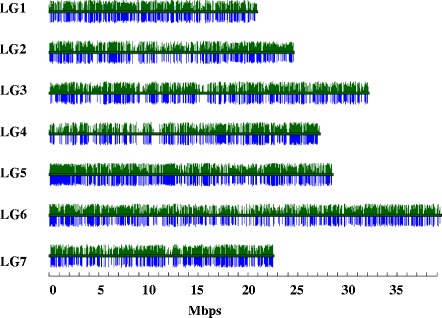
**Distribution of minor allele frequency (MAF ≥ 0.1 in green, ≥ 0.35 in blue) of SNPs across seven LGs.** MAF is shown according to physical location on the *F. vesca ‘*Hawaii 4’ v1.0 reference genome in 65 diverse strawberry accessions.

SNP polymorphism as present in wider germplasm was estimated based on MAF in the 12,609 *PHR* SNPs obtained after filtering across the 65 diverse cultivar accessions (Diversity set, Additional file 1). MAF ranged from 0 to 0.50 with 0 indicating monomorphism and 0.50 indicating the presence of both alleles at equal frequency. Approximately 91% (11,518) of the SNPs were polymorphic as indicated by a MAF ≥0.10. These polymorphic SNPs (MAF ≥0.10), and a highly polymorphic fraction of SNPs with MAF ≥0.35 (4,097 SNPs or 32.5% of the total), were well distributed across the seven pseudochromosomes of the *F. vesca ‘*Hawaii 4’ v. 1.1 reference genome according to physical location (Figure 14). The largest SNP gap was 642 Kbp in length and observed on linkage group 4 (Figure 14). Nine (0.07%) SNPs were monomorphic, while 1,082 SNPs (7.1%) exhibited low polymorphism with an observed MAF <0.10 in the evaluated germplasm.

## Discussion

Implementation of marker-assisted breeding, including genome wide selection and upstream genomics studies such as genome-wide association studies, requires large numbers of robust markers that are widely distributed across the genome. Single nucleotide polymorphisms are the preferred marker type for these applications due to their preponderance in the genome and their amenability to automated genotyping [61,62]. For maximum utility in the allo-octoploid, cultivated strawberry, the genotyping platform must interrogate and provide accurate genotypic information for large numbers of SNP markers that occur in a predominantly octoploid genomic setting and that are distributed across the multiple subgenomes. As part of the RosBREED project [1], a broadly collaborative research and development project was initiated to establish a high-throughput SNP genotyping platform for octoploid strawberry and to assess its usefulness. To this end, multiple approaches were developed and implemented for identifying SNPs suitable for genotyping on an Affymetrix Axiom array platform coupled with the Axiom GT1 clustering algorithm for automated SNP genotype calling. These approaches included the development of multiple bioinformatic pipelines and optimized strategies for SNP discovery and genotyping at the octoploid level.

The tangible product of this initiative is the IStraw90 Axiom array, released commercially by Affymetrix on October 22, 2013. In total, 95,062 marker loci (SNPs, indels, and haploSNPs) were included on the array, of which 85,663 were developed on the basis of discovery in an octoploid germplasm discovery panel, 3,751 were discovered in the important diploid mapping parent *F. iinumae* F1D, and 5,648 were developed via a speculative, non-discovery based approach. In an initial (Phase I) assessment of array performance in two octoploid progeny populations (HK and CCF), a diverse sampling of cultivated and non-cultivated octoploid germplasm, and a diploid mapping population, performance of the array has met or exceeded expectations. Further evaluation and analysis will be required to establish the full potential of the array; however, ongoing improvements in gridding and genotyping algorithms promise to further increase the number of useful markers. In addition to evaluating the marker classes obtained from this new gridding algorithm, the array will be evaluated in additional strawberry germplasm. Still, the substantial demand for the array that has already arisen internationally in strawberry genomics and breeding communities is evidence of the need for such a tool and the platform’s realized effectiveness.

### Performance of the array in linkage mapping

Of the 95,062 marker loci on the array, it has thus far been possible to incorporate 6,594 markers from the filtered and most amenable marker class of 12,609 *PHR* markers into an octoploid linkage map based upon the cross ‘Holiday’ × ‘Korona’. Linkage group segments on the resulting map that most lacked markers usually coincided with chromosomal regions for which ‘Holiday’ and ‘Korona’ are known to be homozygous based on SSR-haplotype information [38]. The genetic map length of 2,050 cM is close to the reported 1,760 cM for the segregating part of the integrated SSR linkage map [38]. In most cases, the lengths of the LGs were similar to those of the published SSR linkage maps of ‘Holiday’ × ‘Korona’, where less than 10 cM differentiated 16 of the 28 chromosome pairs (Table 6). In some cases, however, the lengths of the SNP maps were larger, and in two cases (LG 3B, and 6C) they were at least 50 cM longer. The existing SSR maps well represented the proximal and distal ends of the physical map of the *F. vesca* reference genome [38]. Major increases in sizes of the SNP maps could thus not be due to an actual increase in the represented genomic region, but are likely to be due to genotyping errors for these *PHR* markers, which had not been scrutinized further following filtering. Other aspects that may affect the length of linkage maps are the ease by which the two parental maps could be integrated, in addition to differences in coverage and mapping algorithm. The relative importance of these factors may be evaluated once the SNP data have been carefully examined for other SNP linkage maps.

**Table 6:** **Lengths (in cM) of the 28 integrated genetic linkage groups of the full-sib family ‘Holiday’ × ‘Korona’**

**LG**	**Subgenome**							
	**A**		**B**		**C**		**D**	
	**SSR**	**SNP**	**SSR**	**SNP**	**SSR**	**SNP**	**SSR**	**SNP**
1	42	59	62	61	39	35 + 1^1^	7	12 + 1
2	88	92	79	58 + 11	69	22 + 32	70	76
3	53	76	67	116	73	79	62	94
4	54	49 + 1	83	77	82	6 + 36	62	73
5	78	89	87	60	64	66	79	76
6	84	118	94	114	84	152	74	92
7	76	35 + 34	53	70	8.4	5	61	69

^1^Two SNP sub-maps present (see Figure 11), due to which the total length could not be estimated.

Lengths are based on previous estimation using highly scrutinized SSR markers [38] taking into consideration only the segregating part of these SSR maps, and on current observations using filtered but otherwise non-scrutinized SNP markers.

Ongoing analyses indicate that thousands of additional, non-*PHR* (e.g., *NMH* and *OTV*) markers could be mapped, and thus the full potential of the array for mapping in the ‘Holiday’ × ‘Korona’ cross has yet to be realized. Nevertheless, the existing set of 6,594 markers provides coverage of all 28 expected linkage groups, with an average marker density of approximately one marker per 0.5 cM, thus exceeding our benchmark goal of one marker per cM. Results to date indicate that a similar number of markers can be mapped in the ‘Capitola’ × CF1116 population, and more than 6,000 markers have been identified as robust and suitable for mapping in a four-generation “non-ananassa” pedigree-connected population (unpublished data).

The availability and use of *OTV* SNPs not only increased marker density but also increased the power for integration of the maternal and paternal linkage maps in these F_1_ mapping populations. The introduction of a third null allele in addition to the two conventional alleles A and B introduces markers of AB × AC segregation type through the crosses AB × A∅, AB × B∅, A∅ × B∅, and their reciprocals. Their segregation is fully informative in both parents, whereas SNPs of segregation type AB × AB are informative for only 50% of meioses. Null alleles were also fully informative markers in developing a linkage map in four inter-specific crosses of pear as demonstrated by Montanari *et al.* [4]. Accounting for null alleles introduces bridge markers of the highest level of informativeness. For LG6D, one such SNP marker was present in HK (OTV-7, Figure 10V) as two SSR markers. Scrutinizing genotype calls at the cluster and/or individual sample level may also increase the number of useful markers. This strategy should facilitate the mapping process and improve overall quality of maps, resulting in shorter lengths and more correct marker orders. This improvement was demonstrated for the mapped *PHR*, *NMH*, and *OTV* SNPs of LG6D.

### Success rate & number of informative and high quality SNPs

In addition to the 6,594 *PHR* markers mapped in HK, another 30K SNPs of similar quality were polymorphic with other test panel germplasm, but could not be genetically mapped because they were monomorphic in the HK family. This success rate, which is much higher than expected, is likely driven by the greater than expected presence of naturally occurring variation in effective ploidy level within polyploid individuals, as well as by the array design employed and SNP genotyping strategies.

At face value, the success rate expressed as a proportion of the 90K markers on the array that were mapped in a population is comparatively low; however, this rate was expected in part due to the challenges of octoploidy for genotype calling. In addition, the success rate was expected to be lowered because a substantial proportion of array space was invested in exploring innovative strategies, the outcomes of which are expected to inform future array development efforts in strawberry and other polyploids.

### Synteny and divergence among subgenomes

High levels of synteny among the four subgenomes have been demonstrated by conserved order of SSR loci on SSR-based genetic linkage maps (e.g., [38,39]). These same maps demonstrated subgenome divergence as SSR primer pairs frequently generated amplicons for only some of the four subgenomes. Diversification was also demonstrated by the current HK SNP data, where the number of mapped SNP markers tended to decrease from subgenome A to subgenomes C and D (Table 5). This pattern presumably reflects sequence divergence between the latter two subgenomes and the reference *F. vesca* genome. Such divergence may explain why up to 50% of the Illumina reads from the re-sequenced octoploid strawberry cultivars could not be aligned to the reference genome (data not shown). Physical maps for additional progenitor *Fragaria* species are needed to better represent the genetic diversity between the subgenomes of *F. ×ananassa*.

A central and recurring issue for array development in a polyploid organism is the relationship between ploidy reduction – both technical and biological – and cluster compression. The cultivated strawberry and its immediate ancestors *F. chiloensis* and *F. virginiana* are octoploids, as defined by chromosome number (2*n* = 8*x* = 56), and so the default expectation was that any given SNP locus would be represented eight times in the genome (two alleles in each of four subgenomes). Thus, in principle, genotyping of a target SNP segregating in only one subgenome would have to be successfully achievable in an octoploid context wherein an identified marker allele would be present in two, one, or zero copies in combination, respectively, with six, seven, or eight background alleles. Thus, the marker genotypes to be differentiated by genotyping would be: AABBBBBB, ABBBBBBB, and BBBBBBBB, where A is the “marker allele”. The parallel diploid case would be AA, AB, and BB, without the many additional B alleles. Thus, the preponderance of “background” alleles in an octoploid as compared with the diploid setting is the source of excessive signal from the array probe(s), resulting in cluster compression (Figure 4). Cluster compression is the primary challenge to genotyping in any allo-polyploid and particularly in an octoploid, and is an issue for all genotyping platforms such as Illumina Infinium and Affymetrix Axiom platforms that rely on two-color probe labelling systems.

Ploidy reduction is a potential solution to the problem of cluster compression. In the present study, we devised and successfully implemented strategies for “technical” ploidy reduction, in which three categories of haploSNP sites were identified and targeted with probes intended to have subgenomic specificity.

### Biological ploidy reduction

**Figure 15:**
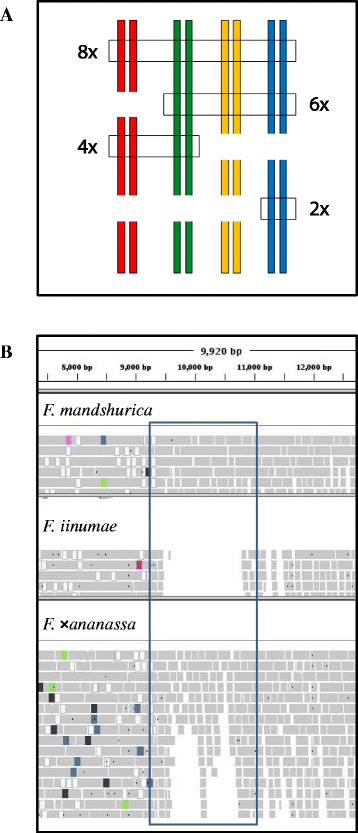
**Genomic basis for biologically effective ploidy reduction. A**. Site-specific ploidy reduction in one or more subgenomes is a proportional consequence of site-specific deletion within the alternate subgenomes. Site-specific ploidy may be reduced from the octoploid (8*x*) to the hexaploid (6*x*), tetraploid (4*x*), or diploid (2*x*) levels. **B**. Alignment of Illumina short reads to the ‘Hawaii 4’ v1.1 reference genome reveals a ~1.5 kb region of localized read depth reduction, indicative of ploidy reduction, in octoploid *F. ×ananassa* ‘Winter Dawn’, corresponding to the site of a ~1.5 kb deletion in diploid *F. iinumae* relative to the *F. vesca* diploid reference genome. The deletion is absent in diploid *F. mandshurica*, a close relative of *F. vesca*. Visualized in Integrated Genome Viewer (IGV, Broad Institute).

In addition to “technical ploidy reduction”, our outcome benefitted from “biological” ploidy reduction, which derived from the fortuitous existence of localized regions of effectively reduced ploidy in an otherwise octoploid genomic context, with the ploidy reduction assumed to be the result of large or small scale insertions and deletions among subgenomes (Figure 15).

The existence of localized regions of reduced ploidy is indicated by several factors. First, the size (C value) of the octoploid genomes is almost 25% less than the expected four times the size of the average diploid strawberry genome [63], of which the ~260 Mb flow cytometric size of the ‘Hawaii 4’ genome is typical. Thus, genomic DNA loss has evidently occurred since the origin of the octoploids as observed in numerous polyploid species (e.g., [64]). One manifestation of such loss is the reduction in number of 25S and 5S rRNA sites in octoploid *Fragaria* species as detected by FISH [65]. Also, sites of localized read depth reduction were evident in alignments of octoploid read sets to the reference genome, and at least some of these sites coincided with sites of species-specific deletion in the ancestral diploid species, as exemplified by *F. iinumae* (Figure 15). In addition, plotting of mean read depths at gene sites throughout octoploid genomes revealed a distribution that is far from normal (Figure 9), with multi-modality suggestive of underlying partitioning into distinct classes. These classes might correspond to genomic regions that are effectively diploid, tetraploid, hexaploid, and (predominantly) octoploid. Finally, a substantial proportion (32.2%) of the 10,072 filtered *PHR* standard SNPs and indels displayed diploid-like clustering, which likely contributed to their *PHR* status. When superimposed on a plot of read depth frequency in relation to mean read depth (Figure 9), the standard SNPs displaying diploid-like clustering were found to occur preferentially at sites of comparatively low read depth, suggesting that these were sites of biologically reduced ploidy, although not necessarily reduced to the diploid level. These could also be sites of high sequence diversification, resulting in homoeolog-specific impairment of probe hybridization. Therefore, in the following discussion of various technical ploidy reduction strategies, the possibility must be recognized that SNP sites targeted for technical ploidy reduction strategies might also correspond to sites of biological ploidy reduction.

### Approaches for technical ploidy reduction

Technical ploidy reduction was sought by means of four SNP discovery strategies. First, SNPs classified as multi-allelic (mSNPs) were thought to hold potential for technical ploidy reduction on the basis that one subgenome might contain alleles that were not represented in the other homeologs (Figure 1B and C) and that with appropriate probe design these segregating marker alleles could be genotyped effectively. However, of all the employed strategies, the mSNP strategy was perhaps the least effective, for several reasons. First, the number of identified candidate sites was relatively few (1,940). Second, the number of probes (four for one strand and eight for two strands) required to interrogate an mSNP site was higher than for any other marker class, thus consuming disproportionate array space. Third, the SNP genotyping algorithm was not, and is still not, optimized for mSNPs, and requires further manual examination. Finally, the conversion rate of mSNPs as measured by the extent of their representation in the *PHR* category was low (Table 4, Figure 7). Conversion rate of mSNPs into filtered *PHR* was ~12.6%. Since each mSNP required, on average, 3.8 times as many probes as di-allelic SNPs (assuming the later were tiled on the same number of strands), targeting mSNPs is not recommended from an efficiency standpoint.

A second and much more effective strategy was the haploSNP approach, which took three distinct forms: a marker SNP nearby a subgenome-differentiating SNP (SNP-SNP; Figure 2A); nearby an indel (Indel-SNP; Figure 2B); or within an insertion (SNP-in-Insertion; Figure 2C). The bioinformatics pipelines needed to identify such sites were somewhat elaborate and case-specific, and while effective were not subjected to extensive optimization. Thus, the potential effectiveness of these pipelines in maximizing the discovery of favorable genomic sites cannot be precisely assessed. Nevertheless, the relative rates of conversion of the three haploSNP forms, as indicated by their participation in the *PHR* class, offers useful insight. By far, the SNP-SNP strategy was the most effective haploSNP strategy, based on two criteria. First, the greatest numbers of haploSNP candidates were in the SNP-SNP class and are therefore more abundant to target (7,764 of the 15,622 candidates submitted to Affymetrix, Table 3). Second, the highest conversion rate into filtered *PHR* (27.2%) was obtained in the SNP-SNP category (Figure 7).

### Transferability to diploid *F. iinumae*

Although the IStraw90 array was developed primarily as a tool for genotyping octoploid strawberry germplasm and breeding materials, for purposes of comparison a set of SNPs was discovered and then genotyped in an important family of diploid individuals, specifically the *F. iinumae* mapping parents and 21 F_2_ generation progeny. Of the 3,751 F1D SNPs, 3,031 or 82% could be incorporated into an *F. iinumae* linkage map. In contrast, of the 85,663 array loci that were based upon discovery in the octoploid germplasm panel, only 199 could be placed upon the *F. iinumae* linkage map, while less than 1% of the 3,751 F1D SNPs achieved the *PHR* rating in relation to octoploid genotyping. Thus, transferability of discovery-based SNP markers between octoploid and diploid germplasm sets in *Fragaria* was very low, suggesting caution as to the applicability of the IStraw90 array for studies in *Fragaria* germplasm other than the octoploids and diploid *F. iinumae*.

### The codon-based strategy

The codon-based strategy was explored as an intriguing option made possible by the large number of SNPs (90K) that could be tested on the Affymetrix Axiom array. Here, the rationale was to test a strategy for developing polymorphic SNPs based on physical location without the need for previously obtained data on sequence variation. However, the conversion rate to useful polymorphism of 5,648 codon-based SNPs on the array was very low (<1%), with zero and seven codon-based markers being incorporated into the HK and F2D linkage maps respectively. Further analysis of these data is in progress, and may reveal opportunities for modification and improvement of the codon-based strategy.

### Array design in allo-octoploids

For array design in allo-polyploids of high ploidy level, a combination of targeting both standard, di-allelic SNPs and ploidy-reducing haploSNPs may be most effective, as these two SNP categories may be complementary with respect to their patterns of genomic distribution. The former may well represent regions of reduced effective ploidy due to true local ploidy reduction or to sequence diversification. The latter may better represent regions that remain at high effective ploidy levels. Furthermore, given the reported trend of biased patterns of gene loss/retention post polyploidization [66], combining standard di-allelic and ploidy-reduction SNPs will likely target genes from different functional categories, useful for future discovery of marker-trait relationships. Functional analyses across the asterids, rosids, and monocots recently confirmed that, post polyploidization, genes involved in “biological regulation” were retained in multiple copies (or were resistant to fractionation) while those responsible for metabolic activities tended to lose copies [66].

## Conclusions

The Affymetrix IStraw90 Axiom array is the first high-throughput genotyping platform for allo-octoploid strawberry. In the design of the array, strategies were successfully developed and applied that enhanced cluster resolution by achieving technical ploidy reduction. The most effective strategy was “SNP-SNP”, in which a subgenome-specific SNP located within 6 bp of a marker SNP was exploited as a probe destabilization site. Presence of diploid-like clusters even in the standard di-allelic SNP category indicated that effective ploidy levels have already been reduced in the octoploid strawberry at multiple genomic regions due to subgenome sequence diversification and subgenomic deletions. Genotyping procedures for polyploids were improved by the addition of new functionalities to the Axiom Best Practices Genotyping Workflow, which streamlined automatic genotyping for compressed clusters and for complex clustering patterns. Validation of the array indicated that combining standard and ploidy-reducing haploSNPs is a useful approach for high-density genome scans and linkage mapping of allo-polyploids of high ploidy levels.

## Additional files

Additional file 1:
**List of 306 cultivated strawberry** ***F.*** **×** ***ananassa*** **samples evaluated with the strawberry array.** This list contains 302 unique accessions because we included four replicate samples of ‘Korona’ and two of ‘Holiday’. Cultivar names and parents are provided. Individuals whose genotyping call rates were below the Affymetrix threshold of 97% and were excluded from genotyping are indicated. Also the sixty-five diverse cultivars used for estimating minor allele frequency are indicated. Additional file 2:
**Illumina library preparation from strawberry GDP.**
Additional file 3:
**Di-allelic SNP discovery pipeline.** Initially, only the “BothSafe” candidates were processed through steps 4–7. Later, when more SNP candidates were needed to fill out the array, the UpSafe candidates were also advanced and utilized. It is noteworthy that step 1, as implemented, excluded sites in which the reference allele differed uniquely from the alleles represented in the discovery panels. For instance, in Figure 1A, cases 1 and 2, if the reference allele had been “C”, which was not represented in any of the discovery panel reads, the variant type would have been reported as *snp,snp,* and would have been classified as multi-allelic and therefore excluded from the di-allelic SNP category. Additional file 4:
**Multi-allelic SNP discovery pipeline.** Five of the eight steps in this pipeline are in common with the di-allelic SNP pipeline (Additional file 3), but are applied in a differing order with the aim of reducing computational time. Only the BothSafe candidate sites were advanced through Steps 4–8 to allow for the option of probing on both strands as needed to resolve the alternate possible genotypes. The mSNP filter pipeline presented some unique challenges, because multiple variants were sought at a single position. The rationale for implementing a “Minimum variant read count” filter twice in the pipeline (Steps 1 and 6) is as follows. Steps 1 and 2 were applied in an integrated process that yielded candidate sites at which at least three reads contained a variant base, but – importantly – the three reads were not required to contain an identical variant at the respective site. Nonetheless, only a small fraction of these sites contained multiple variants, thus explaining why the number of candidate sites dropped so substantially upon the integrated application of steps 3 and 4. At step 6, a minimum read count filter was applied alone to assure that any particular variant (at a given site) was present in at least three reads. Thus, some additional sites were excluded because no one variant (out of the multiple variants present at the site) was present in at least three reads. Thus, the distinction is that at steps 1 + 2, the filter combination was acting to identify qualifying sites, while at step 6 the employed filter was acting to assure that the selected sites contained qualifying variants. Additional file 5:
**Di-allelic indel discovery pipeline.** For indels, the variant read count filter (step 2) was set at x = 2 (rather than x = 3 as used for SNPs) because of the reduced likelihood that indel variants, and especially those of greater than 1 bp would be due to sequencing errors. The UpSafe-DownSafe filters were used to assure that the regions 24 bp upstream and 30 bp downstream of the indel site were free of other variants. The 30 bp (rather than 24 bp) downstream exclusion was required here because the indel site location is defined at a single, upstream reference coordinate, yet it spans several (3 to 6) bp. At step 5, the Genic rather than the CDS filter was employed to enable consideration of indels within introns as well as coding sequences, thus increasing the available number of indel candidates yet avoiding potentially poorly conserved intergenic space. Additional file 6:
**SNP-SNP discovery pipeline.** In pathway step 3, SNPs that can serve as subgenome-specific “destabilization” sites are identified. These SNPs must be present in all 10 HD-16 members. Step 4 identifies instances where a potential marker SNP site is present within 6 bp of an identified “destabilization” site. Steps 5 and 6, including the “SNP Association Check” depicted next, are intended to ensure that the marker SNP is polymorphic only in the subgenome to which the designed probe will be specific. Additional file 7:
**Indel-SNP filter pipeline.** In parallel with the SNP-SNP pipeline (Additional file 6), the purpose of step 3 in the present pipeline is to identify potential subgenome-specific “destabilization” sites, which in the present case are indels rather than SNPs. The maximum size of indels reported in the VCF files is 6 bp; therefore, the minimum size limit imposed at step 3 means that the candidate indels must be in the range of 4–6 bp. Steps 4 through 7 in the present pipeline are analogous to steps 3 through 6 in the SNP-SNP pipeline (Additional file 6), in a differing order. In the present pipeline, the 24 bp +/− filter was not applied because it would have reduced the number of candidate to a negligible level. Details of step 6 and 7 are provided on the second page of this file. Additional file 8:
**SNP-in-Insertion filter pipeline.** Because of the constraints imposed by VCF structure, two distinct pipelines were needed to identify SNP-within-insertion sites. In Case 1 (above, and page 2 of this file), the reference sequence contains the “deletion form” at the site in question. In Case 2 (above, and page 3 of this file), the reference sequence contains the “insertion form” at the site in question. Thus, these two cases are reported separately in the VCF files, as *ins* and *del* variants, respectively. Additional file 9:
**Diploid F1D SNP filter pipeline.** In steps 1 through 3, this pipeline parallels that used for identifying di-allelic SNPs in the octoploid GDP (Additional file 3), a key difference being that only a single VCF file was mined for F1D SNP discovery, while 20 files were simultaneously mined in the octoploid SNP discovery process. Unlike in the octoploid discovery pipelines, linkage group 0 (LG0) of the ‘Hawaii 4’ reference genome was included in the diploid discovery process; however, no SNPs from LG0 were ultimately included in the array. Additional file 10:
**List of 138,099 probesets used to interrogate 95,063 target sites and their physical location.** Polymorphism in ‘Holiday’ and/or ‘Korona’ and presence on the linkage map shown in Figure 11 are indicated. Also indicated are the 12,609 filtered *PHR* SNPs and their clustering pattern (diploid or polyploid). Additional file 11:
**Conversion rate of di-allelic SNPs interrogated with one versus two strands.**
Additional file 12:
**Effect of increasing NoCall rate with decreasing confidence score.** The grey squares signify no calls. The number of grey squares (no calls) increase from Panel A to B to C with the increasingly more stringent confidence score. A. Confidence Score = 0.15 (default) B. Confidence Score = 0.05 C. Confidence Score = 0.01. Additional file 13:
**Graphical genotyping graphs for ‘Holiday’ from different stages of the mapping process.** Panels A & B relate to step 2 of Figure 10 where the original JoinMap derived *PHR* map of 106 cM for the subset of 75 progeny (A) has been manually re-ordered (B). Panel C presents the map for step 5 where the full SNP data set (*PHR*, *NMH*, *OTV* SNPs and 10 SSRs) were scrutinised for some singletons and a pair of double recombinant SNPs. Each colored row represents a single offspring from ‘Holiday’ × ‘Korona’. Each column represents a SNP marker. A green/blue transition within a row indicates a recombination event. Non-colored segments indicate non-informative data, which can be due to true missing data or to non-informative AB genotypes. Pink indicates singletons or a pair of recombinant SNPs. Orange lines indicate unstable map regions. Additional file 14:
**Data underlying the genetic and physical map of LG6D and as used for Figures** **10** **V,** **11** **and** **12** **.** Identity, SNP-class, genetic and physical positions, and JoinMap calls for the ‘Holiday’ x ‘Korona’ progeny. Additional file 15:
**Cluster plots for three** ***OTV*** **s added to LG6D lacking one homozygote cluster (** ***NMH).*** They mapped on LG6D (Figure 10, markers *OTV*-4, −5, −6 respectively). Progenies and their parents ‘Holiday’ and ‘Korona’ are marked by red (∆), green (∆) and blue (∇) triangles respectively. Non-colored triangles represent the other genotyped germplasm. For ‘Holiday’, two replicated samples are presented. The direction of the crosses was confirmed by genetic mapping whereby these *OTV*-*NMH* SNPs integrated well as maternal marker into the *PHR* framework map (Figure 10).

## Abbreviations

BAM: Binary Alignment Map
x: Burrows-Wheeler aligner
CCF: Capitola’ × CF1116
*CRBT*: *CallRate Below Threshold*
GATK: Genome analysis toolkit
GDP: Global Discovery Panel
GT1: Genotyping Algorithm v1
HD-16: High Depth filtration subset with a minimum 16× coverage depth
HD-20: High Depth filtration subset with a minimum 20× coverage depth
HK: Holiday’ × ‘Korona
HomRO: Homozygous Ratio Offset
HSVs: Homoeologous Sequence Variants
LG: Linkage Group
MAF: Minor Allele Frequency
*MHR*: *MonoHighResolution*
mSNP: multi-allelic SNP
*NMH*: *No Minor Homozygote*
*OTV*: *Off-Target Variant*
*PHR*: *Poly High Resolution*
PSVs: Paralogous Sequence Variants
QC: Quality Control
QTL: Quantitative Trait Locus
sai: sequence alignment index
SAM: Sequence Alignment Map
SNP: Single Nucleotide Polymorphism
SRA: Sequence Read Archive
SRX: SRA Experiment Accessions
VCF: Variant Call Format
