# Supplementary material for: Development and preliminary evaluation of a 90 K Axiom® SNP array for the allo-octoploid cultivated strawberry Fragaria × ananassa
Source: BMC Genomics. 2015 Mar 7;16(1):155. doi: 10.1186/s12864-015-1310-1 (PMC4374422; doi:10.1186/s12864-015-1310-1)
Supplement: Additional file 2: — Illumina library preparation from strawberry GDP. [file 12864_2015_1310_MOESM2_ESM.pdf]

Illumina library preparation

We used manufacturer's recommendations for the four libraries prepared with the TruSeq kit. For the libraries prepared using the modified paired-end protocol, 2µg of DNA were sheared using Diagenode's Biorupter XL water sonicator system (Sparta) for 10 to 12 minutes, to produce 300 – 600 bp fragments, and library construction was as previously described [2]. PCR was used to selectively enrich those DNA fragments that had adaptor molecules on both ends, and to amplify the insert library. Each library was independently amplified for 18 cycles using Phusion DNA Polymerase (Finnzymes) and custom-made PCR primers, in place of the standard Illumina PCR primers. The amplified libraries were purified with the QIAquick PCR Purification Kit. The samples were quantitated with Qubit at the Center for Genome Research and Biocomputing (CGRB), at Oregon State University (OSU). Library size was confirmed with the Agilent 2100 Bioanalyzer (Agilent Technologies) at the CGRB. Libraries from each of the three *F. vesca* samples (Table 1) were pooled in equimolar amounts to yield a total oligonucleotide concentration of 10 nM. The libraries were denatured and then processed with the Illumina Cluster Generation Station, following manufacturer's recommendations. The Illumina Genome Analyzer IIx at the CGRB was used for paired-end (PE) sequencing of 80 bp in the *F. iinumae* (CFRA 1849, aka J4) and *F. mandshurica* (CFRA 1947) samples and at *Centre* Nacional d'Anàlisi Genòmica (*CNAG*) for 150 bp PE sequencing of the Dover × Camarosa F2_34 sample, while the HiSeq2000 was used for PE sequencing of 100 bp for the remaining strawberry samples sequenced at the CGRB (Table 1). The HolKor 2637 sample was sequenced twice: once by Illumina and once at the CGRB.

Library preparation and sequencing of the *F. iinumae* F1D sample were performed by the University of Illinois Genome Center using a HiSeq 2000 platform and providing 100 bp PE reads.
