# Supplementary material for: Development and preliminary evaluation of a 90 K Axiom® SNP array for the allo-octoploid cultivated strawberry Fragaria × ananassa
Source: BMC Genomics. 2015 Mar 7;16(1):155. doi: 10.1186/s12864-015-1310-1 (PMC4374422; doi:10.1186/s12864-015-1310-1)
Supplement: Additional file 3: — Di-allelic SNP discovery pipeline. Initially, only the “BothSafe” candidates were processed through steps 4–7. Later, when more SNP candidates were needed to fill out the array, the UpSafe candidates were also advanced and utilized. It is noteworthy that step 1, as implemented, excluded sites in which the reference allele differed uniquely from the alleles represented in the discovery panels. For instance, in Figure 1A, cases 1 and 2, if the reference allele had been “C”, which was not represented in any of the discovery panel reads, the variant type would have been reported as snp,snp, and would have been classified as multi-allelic and therefore excluded from the di-allelic SNP category. [file 12864_2015_1310_MOESM3_ESM.pdf]

Di-allelic SNP

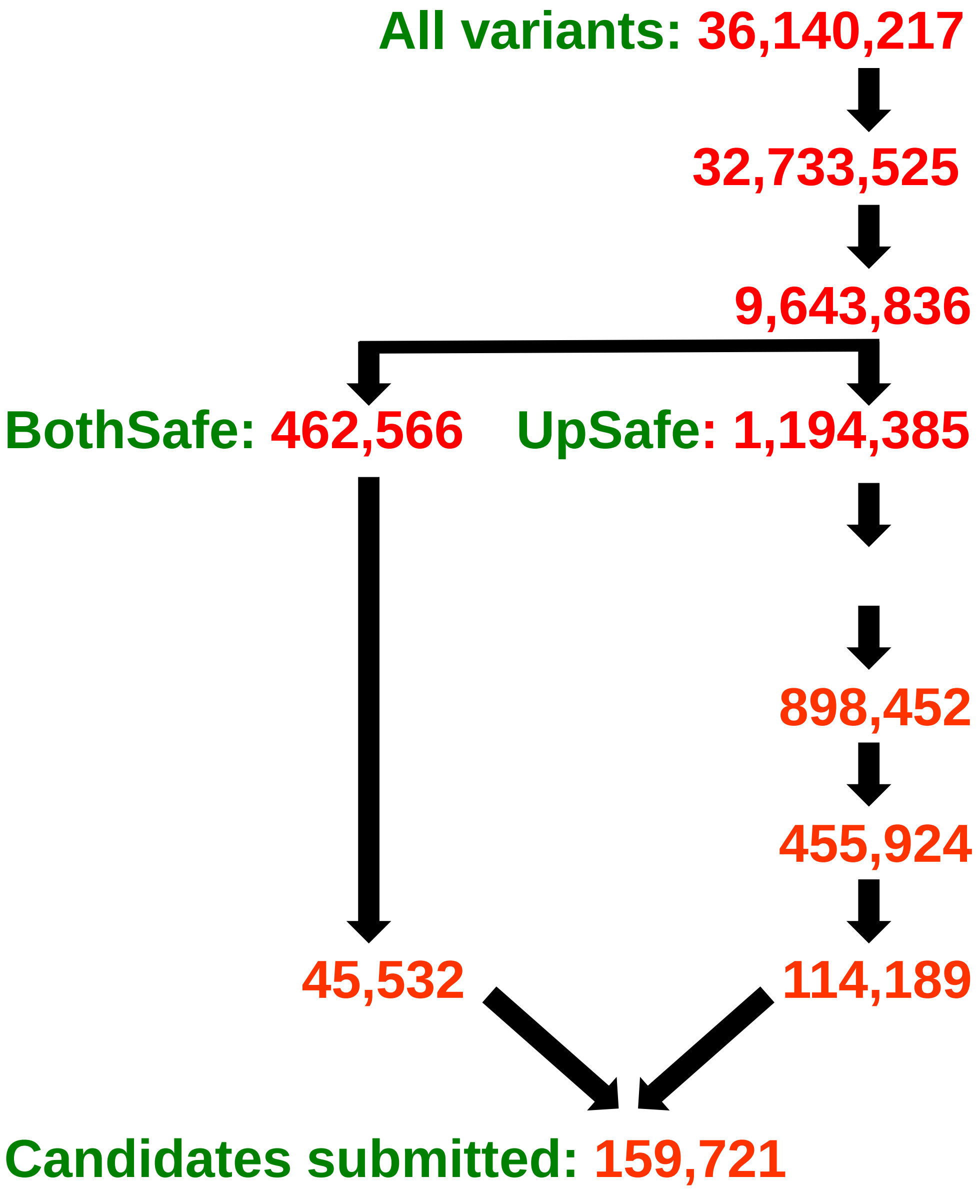

Pipeline steps

1. Retain only *snp* variants.

2. Min. variant read count (x=3).

3. UpSafe-DownSafe (24 +/-).

4. A/T – G/C exclusion.

5. Min. GDP presence (y = 2).

6. Min. HD-20 absence (z = 2).

7. CDS
