# Supplementary material for: Development and preliminary evaluation of a 90 K Axiom® SNP array for the allo-octoploid cultivated strawberry Fragaria × ananassa
Source: BMC Genomics. 2015 Mar 7;16(1):155. doi: 10.1186/s12864-015-1310-1 (PMC4374422; doi:10.1186/s12864-015-1310-1)
Supplement: Additional file 4: — Multi-allelic SNP discovery pipeline. Five of the eight steps in this pipeline are in common with the di-allelic SNP pipeline (Additional file 3), but are applied in a differing order with the aim of reducing computational time. Only the BothSafe candidate sites were advanced through Steps 4–8 to allow for the option of probing on both strands as needed to resolve the alternate possible genotypes. The mSNP filter pipeline presented some unique challenges, because multiple variants were sought at a single position. The rationale for implementing a “Minimum variant read count” filter twice in the pipeline (Steps 1 and 6) is as follows. Steps 1 and 2 were applied in an integrated process that yielded candidate sites at which at least three reads contained a variant base, but – importantly – the three reads were not required to contain an identical variant at the respective site. Nonetheless, only a small fraction of these sites contained multiple variants, thus explaining why the number of candidate sites dropped so substantially upon the integrated application of steps 3 and 4. At step 6, a minimum read count filter was applied alone to assure that any particular variant (at a given site) was present in at least three reads. Thus, some additional sites were excluded because no one variant (out of the multiple variants present at the site) was present in at least three reads. Thus, the distinction is that at steps 1 + 2, the filter combination was acting to identify qualifying sites, while at step 6 the employed filter was acting to assure that the selected sites contained qualifying variants. [file 12864_2015_1310_MOESM4_ESM.pdf]

Multi-allelic SNP

Pipeline steps

All variants: 36,140,217

↓ **1.** Min. variant read count (x = 3).

↓ **2.** All unique variant positions.

Candidate positions: 10,619,615

↓ **3.** UpSafe-DownSafe (24 +/-).

↓ **4.** Multi-allelic SNPs – BothSafe.

18,748

↓ **5.** CDS.

5,947

↓ **6.** Min. variant read count (x = 3).

1,976

↓ **7.** Min. HD-16 presence (y = 2).

1,962

↓ **8.** Min. HD-20 absence (z = 2).

Candidates submitted: 1,940
