# Supplementary material for: Development and preliminary evaluation of a 90 K Axiom® SNP array for the allo-octoploid cultivated strawberry Fragaria × ananassa
Source: BMC Genomics. 2015 Mar 7;16(1):155. doi: 10.1186/s12864-015-1310-1 (PMC4374422; doi:10.1186/s12864-015-1310-1)
Supplement: Additional file 5: — Di-allelic indel discovery pipeline. For indels, the variant read count filter (step 2) was set at x = 2 (rather than x = 3 as used for SNPs) because of the reduced likelihood that indel variants, and especially those of greater than 1 bp would be due to sequencing errors. The UpSafe-DownSafe filters were used to assure that the regions 24 bp upstream and 30 bp downstream of the indel site were free of other variants. The 30 bp (rather than 24 bp) downstream exclusion was required here because the indel site location is defined at a single, upstream reference coordinate, yet it spans several (3 to 6) bp. At step 5, the Genic rather than the CDS filter was employed to enable consideration of indels within introns as well as coding sequences, thus increasing the available number of indel candidates yet avoiding potentially poorly conserved intergenic space. [file 12864_2015_1310_MOESM5_ESM.pdf]

Di-allelic Indel

All variants: 36,140,217
↓
2,135,409
↓
1,193,870
↓
↓
UpSafe+DownSafe+BothSafe: 46,009
↓
17,812
↓
Candidates submitted: 12,801

Pipeline steps

1. Indels only (1 to 6 bp): *ins* or *del.*

2. Min. variant read count (x = 2).

3. UpSafe-DownSafe (24 + / 30 -).

4. Indel size ≥ 3.

5. Genic.

6. Min. HD-20 absence (z = 2).
