# Supplementary material for: Development and preliminary evaluation of a 90 K Axiom® SNP array for the allo-octoploid cultivated strawberry Fragaria × ananassa
Source: BMC Genomics. 2015 Mar 7;16(1):155. doi: 10.1186/s12864-015-1310-1 (PMC4374422; doi:10.1186/s12864-015-1310-1)
Supplement: Additional file 6: — SNP-SNP discovery pipeline. In pathway step 3, SNPs that can serve as subgenome-specific “destabilization” sites are identified. These SNPs must be present in all 10 HD-16 members. Step 4 identifies instances where a potential marker SNP site is present within 6 bp of an identified “destabilization” site. Steps 5 and 6, including the “SNP Association Check” depicted next, are intended to ensure that the marker SNP is polymorphic only in the subgenome to which the designed probe will be specific. [file 12864_2015_1310_MOESM6_ESM.pdf]

SNP-SNP

Pipeline steps

All variants: 36,140,217

⬇ **1.** Min. variant read count (x=3).

⬇ **2.** SNPs only.

9,643,836

⬇ **3.** "Critical" SNP presence: all HD-16.

1,704,226

⬇ **4.** Identify SNP-SNP pairs in HD-16: "critical" SNP coupled to marker SNP.

1,079,658 Pairs (754,931 critical SNPs)

⬇ **5.** Critical SNP presence/absence check.

954,709

⬇ **6.** SNP-SNP association check.

39,522

⬇ **7.** 24 +/- free of other variants.

Candidates submitted: 7,764
