# Supplementary material for: Development and preliminary evaluation of a 90 K Axiom® SNP array for the allo-octoploid cultivated strawberry Fragaria × ananassa
Source: BMC Genomics. 2015 Mar 7;16(1):155. doi: 10.1186/s12864-015-1310-1 (PMC4374422; doi:10.1186/s12864-015-1310-1)
Supplement: Additional file 7: — Indel-SNP filter pipeline. In parallel with the SNP-SNP pipeline (Additional file 6), the purpose of step 3 in the present pipeline is to identify potential subgenome-specific “destabilization” sites, which in the present case are indels rather than SNPs. The maximum size of indels reported in the VCF files is 6 bp; therefore, the minimum size limit imposed at step 3 means that the candidate indels must be in the range of 4–6 bp. Steps 4 through 7 in the present pipeline are analogous to steps 3 through 6 in the SNP-SNP pipeline (Additional file 6), in a differing order. In the present pipeline, the 24 bp +/− filter was not applied because it would have reduced the number of candidate to a negligible level. Details of step 6 and 7 are provided on the second page of this file. [file 12864_2015_1310_MOESM7_ESM.pdf]

Indel-SNP

Pipeline steps

All variants: 36,140,217

↓ **1.** Min. variant read count (x=3).

↓ **2.** Indels only.

1,193,870

↓ **3.** Indel size >3 bp.

208,460

↓ **4.** Critical indel presence: all HD-16.

7,199

↓ **5.** Identify indel-SNP pairs in HD-16:
“critical” indel coupled to marker SNP.

13,301 Pairs (6,640 critical indels)

↓ **6.** Critical indel presence/absence check.

12,753

↓ **7.** Indel-SNP association check.

Candidates submitted: 2,937

The Indel-SNP Association Check

(+) Ref : the Indel form different from the Reference

cYsN (+ -)	= 0 must be in at least 2 samples	reads sum >=3
cYsY (+ +)		reads sum >=3
cNsN (- -)		
cNsY (- +)		

(-) Ref : the Indel form identical to the Reference

cYsN (+ -)		
cYsY (+ +)		
cNsN (- -)	= 0 must be in at least 2 samples	reads sum >=3
cNsY (- +)		reads sum >=3

This filter is found to be the strongest constraint. It eliminates the majority of the candidates.

Note:
c : Critical Indel; s : Marker SNP

A less stringent setting is used:
2 samples → 1 samples
