# Supplementary material for: Development and preliminary evaluation of a 90 K Axiom® SNP array for the allo-octoploid cultivated strawberry Fragaria × ananassa
Source: BMC Genomics. 2015 Mar 7;16(1):155. doi: 10.1186/s12864-015-1310-1 (PMC4374422; doi:10.1186/s12864-015-1310-1)
Supplement: Additional file 8: — SNP-in-Insertion filter pipeline. Because of the constraints imposed by VCF structure, two distinct pipelines were needed to identify SNP-within-insertion sites. In Case 1 (above, and page 2 of this file), the reference sequence contains the “deletion form” at the site in question. In Case 2 (above, and page 3 of this file), the reference sequence contains the “insertion form” at the site in question. Thus, these two cases are reported separately in the VCF files, as ins and del variants, respectively. [file 12864_2015_1310_MOESM8_ESM.pdf]

SNP-in-Insertion

Case 1:

A G C T G A [_ _ _ _ _] G C T T G (REFERENCE)
A G C T G A [A A C T A] G C T T G
A G C T G A [A A G T A] G C T T G
(INSERTION with respect to reference)

Case 2:

A G C T G A [_ _ _ _ _] G C T T G
(DELETION with respect to the reference)
A G C T G A [A A G T A] G C T T G (REFERENCE)
A G C T G A [A A C T A] G C T T G

SNP-in-Insertion (Case 1)

All variants: 36,140,217

Pipeline steps

1. Min. variant read count (x = 3).

2. Insertion(s) present at variant site.

931,096

3. Insert(s) size(s) >3 bp.

188,146 (107,831 unique positions)

4. Positions with multiple, distinct insertions.

55,399

5. Insertion sites that contain SNPs.

14,430

6. Enumerate insertion pairs that form SNP.

17,129

7. Insertion and deletion forms must have at least 3 reads to support.

4,631

2. Insertion and deletion forms present in all HD-16.

Candidates submitted: 2,743

SNP-in-Insertion (Case 2)

Pipeline steps

All variants: 36,140,217

↓ **1.** Min. variant read count (x = 3).

↓ **2.** Indels only.

1,193,870

↓ **3.** Indel size >3 bp.

208,460

↓ **4.** Critical indel presence: all HD-16.

7,199

↓ **5.** SNP within 14 bp of critical insertion.

19,032

↓ **6.** SNPs within critical insertion.

5,731

↓ **7.** SNP absent in at least one of HD-16.

Candidates submitted: 2,178
