# Supplementary material for: Development and preliminary evaluation of a 90 K Axiom® SNP array for the allo-octoploid cultivated strawberry Fragaria × ananassa
Source: BMC Genomics. 2015 Mar 7;16(1):155. doi: 10.1186/s12864-015-1310-1 (PMC4374422; doi:10.1186/s12864-015-1310-1)
Supplement: Additional file 9: — Diploid F1D SNP filter pipeline. In steps 1 through 3, this pipeline parallels that used for identifying di-allelic SNPs in the octoploid GDP (Additional file 3), a key difference being that only a single VCF file was mined for F1D SNP discovery, while 20 files were simultaneously mined in the octoploid SNP discovery process. Unlike in the octoploid discovery pipelines, linkage group 0 (LG0) of the ‘Hawaii 4’ reference genome was included in the diploid discovery process; however, no SNPs from LG0 were ultimately included in the array. [file 12864_2015_1310_MOESM9_ESM.pdf]

F1D-SNP

Pipeline steps

All variants: 8,373,559

↓ **1.** Exclude indels.

↓ **2.** Min. variant read count (x=3).

6,391,052

↓ **3.** BothSafe (24 +/-) in F1D.

2,261,115

↓ **3.** SNP site read depth ≥ 20.

1,659,117

↓ **4.** SNP ≥ 3 reads.

1,658,845

↓ **5.** Heterozygosity check; ~1:1 ratio alternate alleles.

85,352

↓ **6.** A/T – G/C exclusion.

68,172 (including 3,275 from LG0)

↓ **7.** CDS (except in LG0).

23,190 (19,915 + 3,275)

↓ **8.** BothSafe (24 +/-) in GDP.

17,534

↓ **9.** Exclude redundant codon based.

Candidates submitted: 17,518 (including 3,275 from LG0)
