# Supplementary figures and images for: Development and preliminary evaluation of a 90 K Axiom® SNP array for the allo-octoploid cultivated strawberry Fragaria × ananassa
Source: BMC Genomics. 2015 Mar 7;16(1):155. doi: 10.1186/s12864-015-1310-1 (PMC4374422; doi:10.1186/s12864-015-1310-1)

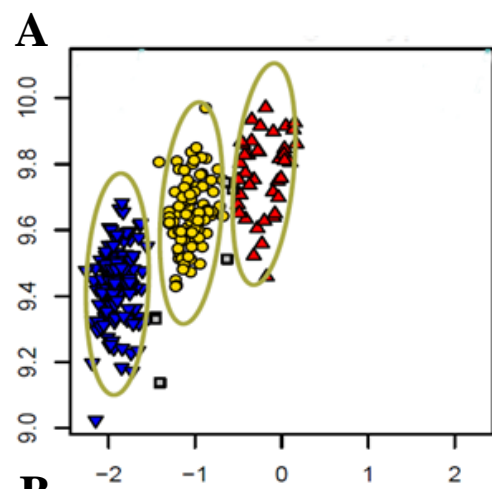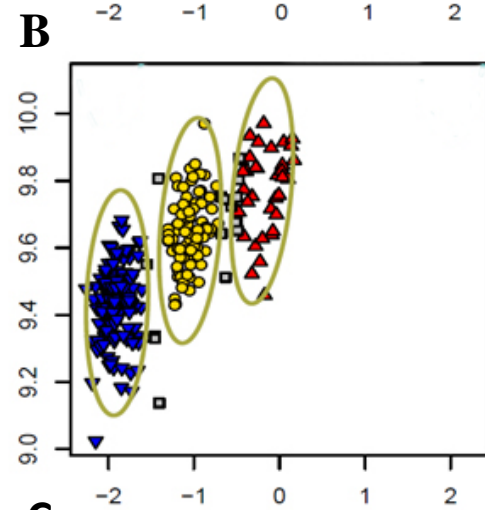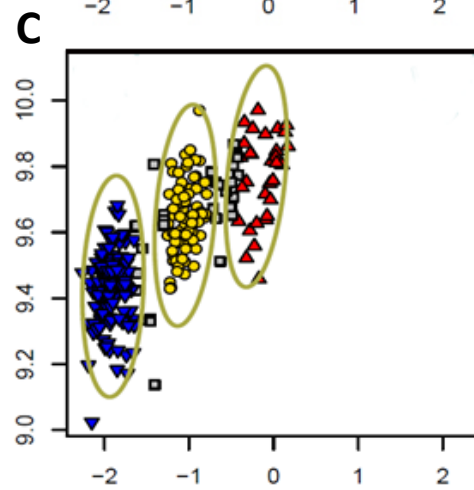

**C**

Supplement: Additional file 12: — Effect of increasing NoCall rate with decreasing confidence score. The grey squares signify no calls. The number of grey squares (no calls) increase from Panel A to B to C with the increasingly more stringent confidence score. A. Confidence Score = 0.15 (default) B. Confidence Score = 0.05 C. Confidence Score = 0.01. [file 12864_2015_1310_MOESM12_ESM.pdf]

**A**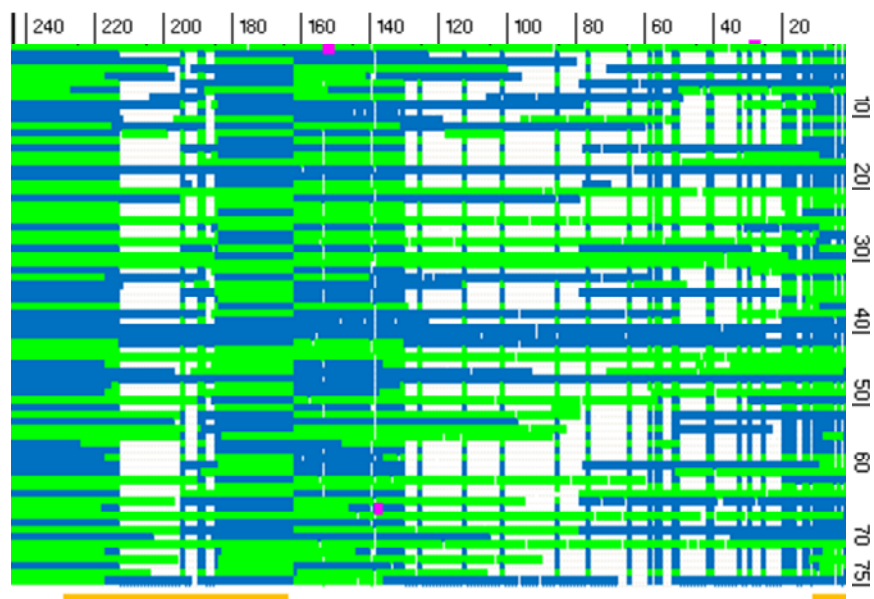**B**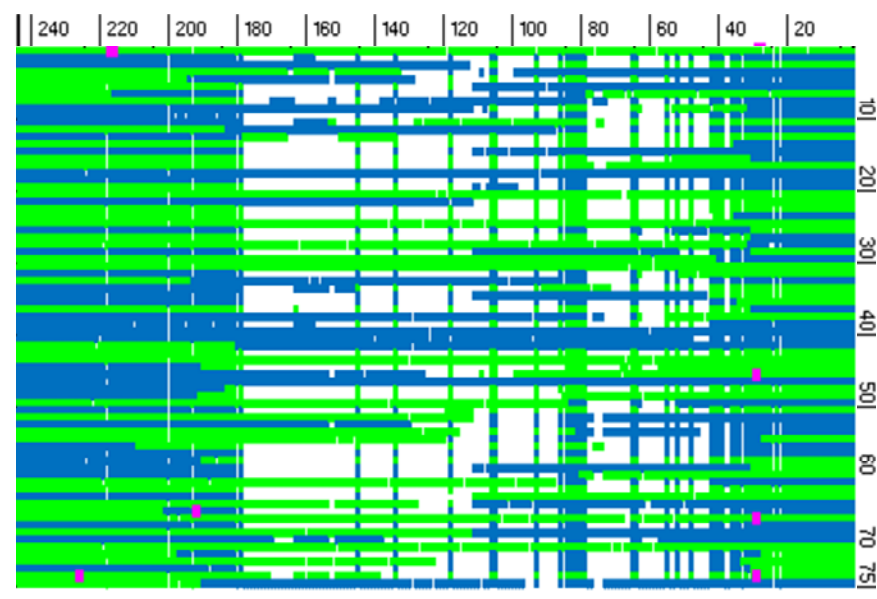**C**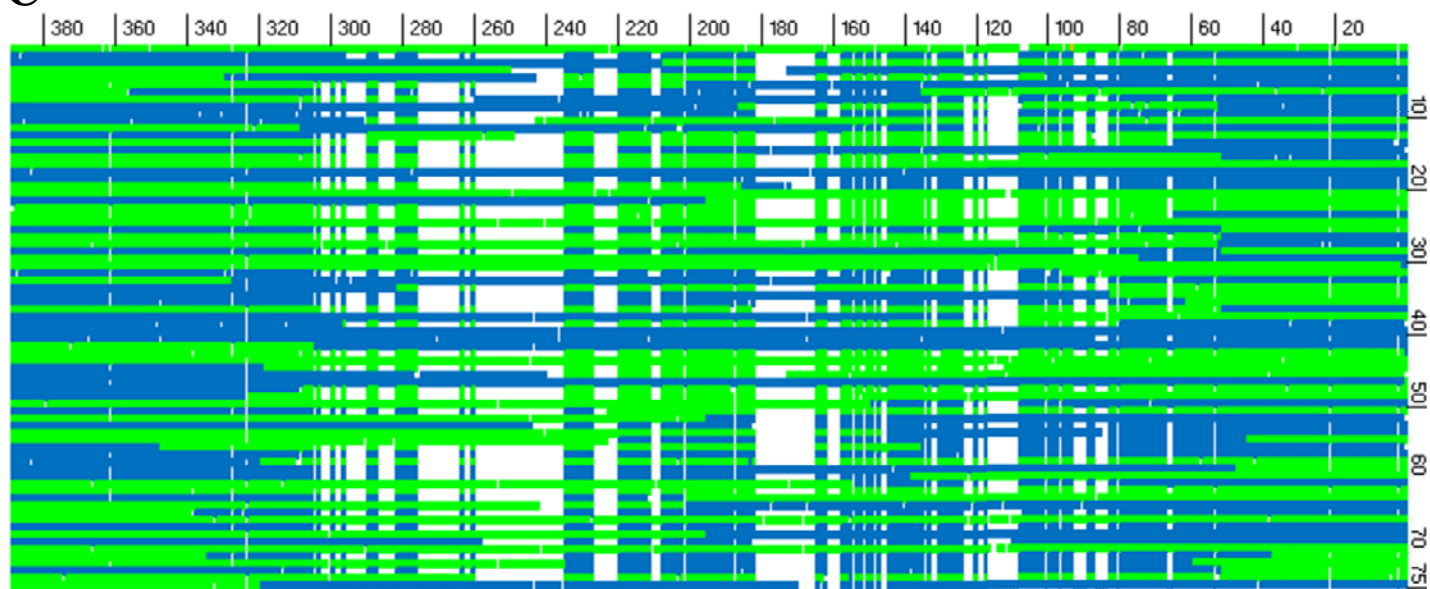

Supplement: Additional file 13: — Graphical genotyping graphs for ‘Holiday’ from different stages of the mapping process. Panels A & B relate to step 2 of Figure 10 where the original JoinMap derived PHR map of 106 cM for the subset of 75 progeny (A) has been manually re-ordered (B). Panel C presents the map for step 5 where the full SNP data set (PHR, NMH, OTV SNPs and 10 SSRs) were scrutinised for some singletons and a pair of double recombinant SNPs. Each colored row represents a single offspring from ‘Holiday’ × ‘Korona’. Each column represents a SNP marker. A green/blue transition within a row indicates a recombination event. Non-colored segments indicate non-informative data, which can be due to true missing data or to non-informative AB genotypes. Pink indicates singletons or a pair of recombinant SNPs. Orange lines indicate unstable map regions. [file 12864_2015_1310_MOESM13_ESM.pdf]

### *OTV-4*

AX-89871559

AvM - called genotypes

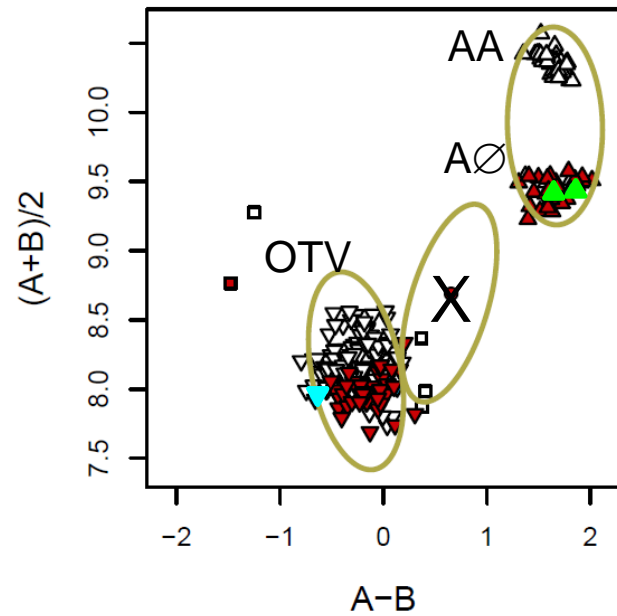

### *OTV-5*

AX-89896961

AvM - called genotypes

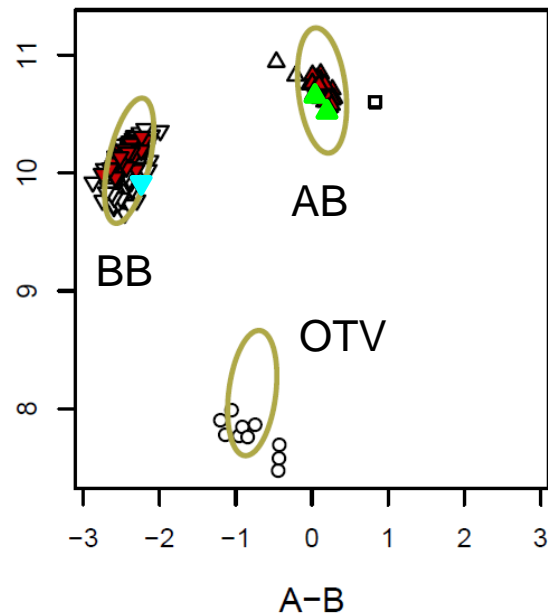

### *OTV-6*

AX-89897092

AvM - called genotypes

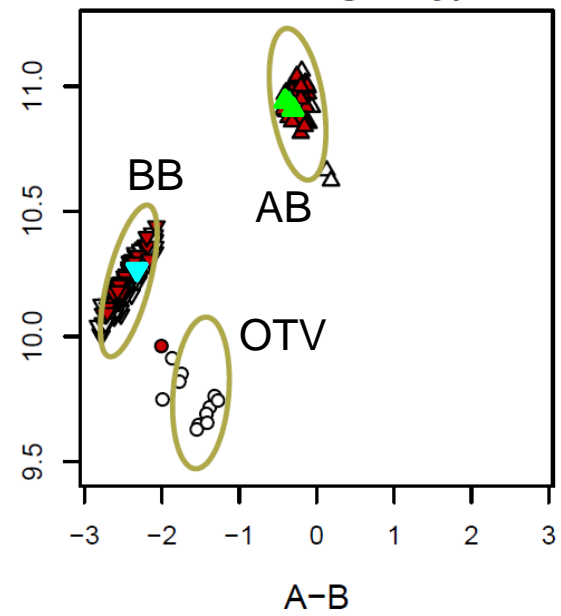

Supplement: Additional file 15: — Cluster plots for three OTV s added to LG6D lacking one homozygote cluster ( NMH). They mapped on LG6D (Figure 10, markers OTV-4, −5, −6 respectively). Progenies and their parents ‘Holiday’ and ‘Korona’ are marked by red (∆), green (∆) and blue (∇) triangles respectively. Non-colored triangles represent the other genotyped germplasm. For ‘Holiday’, two replicated samples are presented. The direction of the crosses was confirmed by genetic mapping whereby these OTV-NMH SNPs integrated well as maternal marker into the PHR framework map (Figure 10). [file 12864_2015_1310_MOESM15_ESM.pdf]
